# Genome sequence of *Phormia regina* Meigen (Diptera: Calliphoridae): implications for medical, veterinary and forensic research

**DOI:** 10.1186/s12864-016-3187-z

**Published:** 2016-10-28

**Authors:** Anne A. Andere, Roy N. Platt, David A. Ray, Christine J. Picard

**Affiliations:** 1 Department of Biology, Indiana University Purdue University Indianapolis, 723 W. Michigan Street, Indianapolis, IN 46202 USA; 2 Department of Biological Sciences, Texas Tech University, Box 43131, Lubbock, TX 79403-3131 USA

**Keywords:** Genome, *Phormia regina*, Calliphoridae, Blow fly, Sex determination, X chromosome, Y chromosome

## Abstract

**Background:**

Blow flies (Diptera: Calliphoridae) are important medical, veterinary and forensic insects encompassing 8 % of the species diversity observed in the calyptrate insects. Few genomic resources exist to understand the diversity and evolution of this group.

**Results:**

We present the hybrid (short and long reads) draft assemblies of the male and female genomes of the common North American blow fly, *Phormia regina* (Diptera: Calliphoridae). The 550 and 534 Mb draft assemblies contained 8312 and 9490 predicted genes in the female and male genomes, respectively; including > 93 % conserved eukaryotic genes. Putative X and Y chromosomes (21 and 14 Mb, respectively) were assembled and annotated. The *P. regina* genomes appear to contain few mobile genetic elements, an almost complete absence of SINEs, and most of the repetitive landscape consists of simple repetitive sequences. Candidate gene approaches were undertaken to annotate insecticide resistance, sex-determining, chemoreceptors, and antimicrobial peptides.

**Conclusions:**

This work yielded a robust, reliable reference calliphorid genome from a species located in the middle of a calliphorid phylogeny. By adding an additional blow fly genome, the ability to tease apart what might be true of general calliphorids vs. what is specific of two distinct lineages now exists. This resource will provide a strong foundation for future studies into the evolution, population structure, behavior, and physiology of all blow flies.

**Electronic supplementary material:**

The online version of this article (doi:10.1186/s12864-016-3187-z) contains supplementary material, which is available to authorized users.

## Background

One in ten species on earth are flies (Diptera). They are the most derived group within arthropods, and have experienced an explosive radiation in the last 50 million years [1, 2]. Over the past decade, dipteran draft genomes including the fruit fly (*Drosophila melanogaster*, [3]), the house fly (*Musca domestica*, [4]), and the malaria mosquito (*Anopheles gambiae*, [5]) have been published. Within Diptera, the family Calliphoridae, commonly known as blow flies, comprises ~1500 species [6, 7], and contributes 8 % of species diversity in calyptrate flies [8]. Calliphorids are ubiquitous, distributed world-wide, and are important in the medical [9–12], veterinary/agricultural [13–16] and forensic fields [17, 18]. For example, blow flies are responsible for the transmission of human disease [19–22]. Mihalyi’s danger-index was calculated for seven blow fly species in South America, with consideration for the synanthropic index. Six of the seven species posed a greater sanitary risk than the house fly [23], a known disease vector [24, 25]. Interestingly, other closely related blow fly species have been shown to be medically advantageous as a means of wound debridement, otherwise known as maggot therapy [9, 12, 26, 27]. In these cases, maggots physically debride the wound of decaying tissue while simultaneously excreting antimicrobial compounds [26, 28–36] effective against antibiotic resistant bacteria such as methicillin resistant *Staphylococcus aureus* (MRSA) [29, 37].

Recently, the first calliphorid genome, from the sheep blow fly (*Lucilia cuprina,* [38]), was released. The publication of the *L. cuprina* draft genome brings with it the potential for studying a group of flies that have evolved recently [1, 2, 39], and have adopted many different life histories [6]. For example, the sheep blow fly specimen(s) that was sequenced was from a location in which it is predominantly, if not exclusively, an obligate ectoparasite (infestation of living vertebrate tissue by fly larvae) [38], and has presumably adapted under selective pressures from subsisting on carrion to infesting live animals. Calliphorid genomes will provide the necessary resources needed to understand the basic biological processes of lineage-specific traits in myiasis-causing flies.

The black blow fly, *Phormia regina* (Meigen), is a Palearctic fly with records throughout North America and Northern Europe, and is the dominant carrion fly for most of Canada and the United States [40]. We chose to examine the genome of this species because 1) it plays an important role in ecosystems via carrion decomposition and nutrient recycling [41], 2) its’ abundance in North America, and 3) because it exhibits no specialized parasitic adaptations or unusual sex determination strategies (i.e. it is not monogenic), though the sex determination strategy for *Phormia regina* is largely unknown. Like other calliphorid flies, *P. regina* contains 2n = 12 chromosomes, including heteromorphic sex chromosomes [42, 43]. These characteristics make it a good candidate for comparisons with species that have more specialized life histories. Furthermore, providing an additional reference genome from Calliphoridae will allow for a more complete understanding of the clade and adaptive processes that take place within it. We sequenced the male and female genomes (~40X), allowing us to characterize sex chromosomes and sex determining pathways, as well as evolutionary relationships of chemoreceptors, antimicrobial peptides, and insecticide resistance pathways in relation to other calliphorids and dipterans.

## Methods

### Genome sequencing – short reads

Genomic DNA was extracted from whole flies using the DNeasy Blood and Tissue DNA Extraction kit (Qiagen Inc., Valencia, CA) and pooled from five female and five male *Phormia regina* flies housed in a laboratory colony (approximately 4–6 months old). The founders originated from Indianapolis, IN (39.7684° N, 86.1581° W) and were collected during the summer of 2012. The extracted DNA from each individual was quantified using a Qubit fluorometer (ThermoFisher Scientific, Grand Island, NY) and mixed in equal proportions to yield the two pooled extracts, one for each sex. DNA libraries were prepared using TruSeq DNA sample preparation (Illumina, San Diego, CA) and sequenced (2 × 100 bp) using one half lane of an Illumina HiSeq2000 platform at the Purdue University Genomics Core Facility (West Lafayette, IN). Additional 454 reads were obtained as described in [44].

### Genome sequencing – long reads

Genomic DNA was extracted from a whole single male *P. regina* specimen that had been in colony for >15 generations (different colony from above, but same originating location, Indianapolis). DNA library preparation and sequencing was performed according to the manufacturer’s instructions and reflects the P6-C4 sequencing enzyme and chemistry, respectively, at the Icahn School of Medicine at Mount Sinai Genomics Core Facility. 14 % of the input library eluted from the agarose cassette and was available for sequencing. For all cases, this yield was sufficient to proceed to primer annealing and DNA sequencing on the PacBio RSII instrument (Pacific Biosciences, Menlo Park, CA). SMRTcell libraries were placed onto the RSII machine at a sequencing concentration of 150 pM and configured for a 240-min continuous sequencing run. Sequencing was conducted to achieve a 7401 bp subread N50 across a total of 1.5 Gb of data comprised of 268,000 reads on 2 SMRTcells.

Due to the high error rate of reads generated from PacBio sequencing [45], error correction was performed using the Correct PacBio Reads tool of CLC’s Genome Finishing Module plug-in v1.5.1 (Qiagen Inc) on a local workstation. Additional error correction was also performed using the SMRT Analysis PacBioToCA correction module, with the assistance of the high quality Illumina reads, using default settings.

### Genome processing and assembly

Genome processing and assemblies were accomplished using CLC Genomics Workbench v6.0.5 (CLC-GWB; Qiagen Inc.). However, in order to evaluate the effectiveness of the CLC genome assembler, preliminary assemblies were generated using *de novo* genome assemblers: Velvet [46] and SOAPdenovo [47]. The CLC assemblies were accomplished using a desktop computer with enhanced memory (32 Gb RAM), whereas the Velvet and SOAPdenovo assemblies were performed using a large memory supercomputing cluster (Indiana University, Bloomington, IN). The male and female Illumina short reads were initially assembled into two draft genomes with the three assemblers. Each assembly was carried out using a range of kmer values until the ‘best’ assembly was captured as determined by contig number and contig N50 size (the kmer values varied with each assembly). The CLC-GWB *de novo* assembly using only Illumina reads resulted in the smallest number of contigs and the longest N50 (Table 1). With this data, we therefore decided to rely solely on CLC-GWB for the remainder of our analyses and any additional assemblies produced using additional reads (454 and PacBio).

**Table 1:** Comparative assembly statistics of preliminary assemblies generated using CLC-GWB, Velvet and SOAPdenovo

Assembler	Sex	Optimal kmer (bp)	# contigs	N50 (bp)	% reads^a^
CLC-GWB	Female	60	251,115	3684	96.0
	Male	60	306,273	2487	93.5
Velvet	Female	75	317,916	1095	47.0
	Male	75	310,045	956	37.9
SOAPdenovo	Female	75	1,953,762	451	68.8
	Male	75	2,010,365	351	58.6

For each assembly pipeline, different kmers were used to generate the optimal assembly, and the main metric for determining quality of assembly was the combination of the number of contigs and the N50

^a^% reads = the number of reads that mapped to each assembled genome

The pipeline for assembly using the Illumina and 454 reads on CLC-GWB was as follows: low quality reads (phred scores < 20) and adaptor sequences were removed, duplicate reads were removed and overlapping pairs were merged (mismatch cost was set to 2 and a gap cost was set to 3). To remove extraneous or contaminating DNA, we used a filtering pipeline that included the mapping and subsequent removal of the above processed reads to 1405 phage (www.phantome.org, downloaded 03/2014) and 595 bacterial genomes (NCBI, www.ncbi.nlm.nih.gov, downloaded 03/2014). Further filtering removed mitochondrial reads, which were assembled separately using methods described below. Approximately 0.38 and 0.49 % of reads were identified as bacteriophage and bacterial contaminants, respectively. Following the removal of these contaminants, 5 % of overlapping paired reads were merged, 1 % of duplicates and low quality reads were removed, leaving a total of 253,233,928 male and 254,306,608 female reads for downstream analyses.

Preliminary assembly iterations involved further optimization of kmer values (ranging from 24 to 60 nucleotides) and bubble sizes (ranging 100–1000 bp). Assemblies were evaluated based on total number of assembled contigs, estimated genome sizes, contig N50 values, and completeness as per CEGMA v2.4.010312 (see below [48]).

Once optimal kmer sizes were determined (45 bp for each individual genome), reads were mapped back to the assemblies using CLC-GWB (mismatch cost of 2, insertion cost of 3, deletion cost of 3, length fraction of 0.5, similarity fraction of 0.8, bubble size 300 bp). PacBio (error-corrected) and 454 reads were combined with the Illumina reads to create a hybrid *de novo* assembly by using them to scaffold the contigs using CLC’s Join Contigs Tool from the genome finishing module plug-in.

#### Mitochondrial genome assembly

The mitochondrial genomes of seven calliphorid flies (*Cochliomyia hominivorax*, NC_002660; *Protophormia terraenovae*, NC_019636.1; *Chrysomya albiceps*, NC_019631.1; *Chrysomya bezziana*, NC_019632.1; *Chrysomya rufifacies*, NC_019634.1; *Chrysomya megacephala*, NC_019633.1; *Lucilia sericata*, NC_009733; *Lucilia cuprina*, NC_019573.1) were obtained from NCBI (www.ncbi.nlm.nih.gov) and used as local reference genomes for the pooled male and female reads. Reads that mapped to these reference genomes were then extracted and assembled using CLC-GWB v7.0.3 to generate a mitochondrial genome assembly. The mitochondrial genome sequence was 99 % similar to a previously published genome from *P. regina* (KC005712, 15,513 bp), 93 % to *Protophormia terranovae* (JX913743.1, 15,170 bp), 92 % to *Chrysomya megacephala* (AJ426041, 15,831 bp), 91 % to *Lucilia sericata* (AJ422212.1, 15,945 bp), and 90 % to *Cochliomyia hominivorax* (AF260826, 16,022 bp), among others. For all calliphorid genomes, sequence similarity was 90 % or greater. All mitochondrial amino acid sequences were 100 % identical to other calliphorid mitochondrial protein sequences.

#### Genome assembly assessment

Assembly statistics were calculated using CLC-GWB and the genome assessment tool QUAST v3.1 [49]. To evaluate the completeness of the genome assemblies, CEGMA v2.4.010312 [48] was used to detect the number of complete and partial core eukaryotic genes present in the assembled genomes. This analysis was completed using GeneWise v2.4.1, NCBI-BLAST+ v2.2.28 and geneid v1.4.4 to return DNA sequences of each prediction and their associated statistical reports.

### Gene prediction & gene ontology

AUGUSTUS [50] was used for *ab initio* prediction of gene sequences based on reference *Drosophila melanogaster* sequences as it is an extensively studied and annotated genetic model organism. Predicted protein sequences were annotated by BLASTp v2.2.28+ using a non-redundant protein BLAST database and an E-value cutoff ≤ 1*e*-5. Comparative analysis of the male and female gene set was performed by using CD-HIT-2D v4.5.6 [51] to compare the two protein datasets (90 % identity, word size (-n) of 5, and length difference cutoff (-s2) of 90 %). The unique protein sequences for the male were blast searched (BLASTp, E-value cutoff ≤ 1*e*-10) against the complete predicted amino acid sequences of the female to confirm their uniqueness. The same was done for the female protein set against the male protein set. Proteins without hits were assumed to be unique to each sex.

Functional characterization of the predicted gene sequences was performed using default settings in Blast2GO v3.1.3 [52]. Gene ontology (GO) terms were assigned to the annotated sequences. GO slim functionality in Blast2GO was used to simplify the annotation into functional categories and the proteins were categorized at level 2 into the three main GO classifications of biological process, cellular component and molecular function. InterProScan [53] statistics and KEGG [54] map pathways were also extracted from Blast2GO v3.1.3 using default values. GO terms from biological processes for each of the unique gene sets were summarized after the removal of redundant GO terms by the web server REViGO (reduce and visualize gene ontology) to create a visual representative subset of the terms using a clustering algorithm that uses similarity measures [55]. The allowed similarity was set to 0.5 (small) and the database of GO terms selected was from *D. melanogaster.*

### Sex chromosome identification

In order to characterize the sex chromosomes, we used the chromosome quotient (CQ) [56] approach which discovers sex chromosome sequences by using a stringent aligning criterion of the male and female reads onto each other’s genomes. The stringent alignment required a whole read to map onto the reference contigs with a zero mismatch in order to reduce the number of false positives [56]. The female to male ratio of the alignments was then used to distinguish contigs from the X or Y chromosome. Male contigs with a CQ of less than the arbitrary 0.05 were grouped as putative Y chromosome contigs and female contigs with a CQ ranging between 1.9 and 2.5 were grouped as putative X chromosome contigs. The sex chromosome contigs were then blast searched against an arthropod database (BLASTn, E-value ≤ 1*e*-5) to determine any homology with other insects in the database. The contigs were also compared against the well-characterized sex chromosomes of *D. melanogaster* (tBLASTx, E-value ≤ 1*e*-5).

### Putative gene approaches

All candidate genes were discovered using one or all of three approaches. First, genes of interest (in particular pathways or associated with potential adaptive traits, see below) were initially acquired from Flybase (www.flybase.org, [57]) using queries for specific genes, or using genes associated with specific GO terms. Contigs with hits were identified via local blast (BLASTn) with an E-value cutoff of ≤ 1*e*-5. Gene sequences were individually annotated for gene structure using the web server version of the AUGUSTUS prediction tool [50], aligned using MUSCLE [58] and viewed with MVIEW [59]. For comparison purposes with other calliphorid or dipteran species, if the gene sequences were available in Genbank, they were acquired and included in our nucleotide (and subsequent predicted amino acid) sequence alignments. If BLASTn approaches of *Drosophila* sequences failed to produce hits, a second approach to discovering candidate genes was to use keyword searches in our annotated gene dataset. A third approach was to use tBLASTx and homologous sequences from other taxa (such as *Lucilia* or *Musca*) on the *P. regina* genomes.

#### Sex-determining genes

Putative sex determining genes were isolated and characterized by querying a set of known genes (*transformer* (*tra*) *–* CG16724, *transformer2* (*tra2*) *–* CG10128, *sex lethal* (*sxl*) *–* CG43770, *doublesex* (*dsx*) *–* CG11094*, fruitless* (*fru*) *–* CG14307*, daughterless* (*da*) *–* CG5102*,* and *maleless* (*mle*) *–* CG11680) from *D. melanogaster* against our male and female assemblies. *tra* and *tra2* gene sequences did not result in contig hits using BLASTn, therefore we used the coding sequences from closely related blow fly species (*Lucilia cuprina –* FJ461621.1 and FJ461620.1*, Cochliomyia macellaria –* JX315619.1*, Cochliomyia hominivorax* – JX315618.1 and *Lucilia sericata –* JX315620.1*)* as well as sequences from other calliphorid genomes (unpublished) for which we have transcriptomic data using discontinuous megablast.

#### Chemoreceptors

*D. melanogaster* gene sequences of odorant binding proteins (OBPs), odorant receptors (ORs), gustatory receptors (GRs) and ionotropic receptors (IRs) were used as query sequences for local blast searches against the *P. regina* genome (E-value cutoff of ≤ 1*e*-5) using tBLASTx (Additional file 1: Table S1). Protein sequences of the ionotrophic receptor *IR25a* were acquired from NCBI from the following species: *Lucilia cuprina-*KNC28739*, Calliphora stygia-*AID61273*, Stomoxys calcitrans-*XP013104244*, Musca domestica-*NP001273813*, Batrocera oleae-*XP014086336*, Ceratitis capitata-*XP004530416*,* and *Drosophila melanogaster-*NP001260049.

#### Antimicrobial peptides

A set of immune-related genes obtained from flybase.org was used to query the *Phormia regina* genome for antimicrobial peptides (attacins, cecropins, defensins, diptericins, Additional file 2: Table S2). A second approach simply queried our BLASTp results (from the predicted) to identify putative immune-related genes that had homologs to other immune-related genes in insects.

#### Insecticide resistance genes

Genes associated with the metabolism of foreign material (xenobiotics) are primarily cytochrome P450′s (Additional file 3: Table S3), glutathione S-transferases (GST, Additional file 4: Table S4) and esterases/hydrolases (Additional file 5: Table S5). These genes were identified by manually searching the BLASTp results from the annotation step of the predicted genes; and KEGG pathways for terms that included cytochrome P450s, GSTs and esterases.

### Repetitive elements

Repetitive elements in the male and female *Phormia regina* genomes as well as the putatively identified X and Y chromosomes were identified using RepeatMasker [60] and a library of all known dipteran transposable elements (TEs; RepBase; accessed 14 March 2015). In addition to known transposable elements, RepeatMasker searches were used to identify low-complexity regions including mini and microsatellite sequences. Output from RepeatMasker was used to quantify overall repeat content and an accumulation profile. For the accumulation profile, which reflects relative rates and periods of TEs in a genome, the Kimura 2-parameter [61] distance between a transposable element insertion and the assumed ancestral sequence were calculated using the calcDivergenceFromAlign.pl script packaged with RepeatMasker.

## Results and discussion

### Genome assembly

Raw reads and genome assemblies have been submitted to GenBank (BioProject ID PRJNA338752, accession numbers MINK00000000.1 and MINJ00000000.1 for the male and female genomes, respectively). Mitochondrial reads (8,378,416) were removed from the main genomic dataset and assembled into a mtDNA genome (15,801 bp, Additional file 6: Figure S1, GenBank accession KX853042).

In order to refine and scaffold our assemblies, we added longer 454 (average read length 344 bp) and error-corrected PacBio reads (average read length 5698 bp). We repeated the *de novo* assembly process in CLC-GWB using a range of kmer values. Our ‘best’ hybrid assembled genomes contained a combination of smaller numbers of contigs and longer N50s (Table 2). The male (534 Mbp) and female (550 Mbp) genomes had average coverages of 44X with >97 % of the reads mapping back to the genomes.

**Table 2:** Final draft genome assembly statistics of the male and female genomes following the addition of 454 and PacBio reads, including a measure of the robustness of the assembly in the number of core eukaryotic genes assembled (CEGMA)

Parameter	Female	Male
Optimal kmer (bp)	60	50
No. of contigs	192,662	187,700
N50 (bp)	7918	7177
Avg. contig (bp)	2859	2846
Max. contig (bp)	116,828	691,679
% reads used	98.03	97.38
Avg. coverage	44X	44X
% AT	72 %	72 %
Genome Size (Mb)	550	534
No. of contigs (> = 1000 bp)	96,027	98,029
Total length Mb (> = 1000 bp)	485	473
No. of N’s per 100 kb	1565	1800
CEGMA % (complete)	93.95	96.77
CEGMA % (partial)	99.19	99.60

These values are larger than the experimentally estimated sizes of 529 Mbp and 517 Mbp for the female and male *P. regina,* respectively [43]. This is likely due to the presence of repetitive sequences that do not assemble well [62], as well as the presence of allelic variation due to pooled sequencing of five male and five female individuals [63]. The robustness of the protein coding portion of the assembly was assessed using CEGMA [48], where 93.95 and 96.77 % of complete core eukaryotic genes were identified in the female and male genomes (see Additional file 7: Table S6 for completeness report).

### Gene prediction and ontology

A total of 9490 and 8312 full length genes were predicted by AUGUSTUS [50] (Table 3) in the male and female genomes, respectively. The genic characteristics of the exonic regions and intronic regions of the predicted genes in both sexes show a total of ~30,000 exons and 20,000 introns (Table 3). The total number of predicted protein-encoding genes in our assembled genomes was small compared to other recently sequenced Dipterans such as *Lucilia cuprina* (14,544 genes) [38] and *Musca domestica* (15,345 genes) [4]. This may be due to the high stringency we used for gene prediction where only complete genes (gene sequences with start and stop codons present within individual contigs) were allowed. With a more contiguous version of the genome, and inclusion of transcriptome data in the prediction process, the predicted gene count will probably increase. For comparison, we predicted complete genes in the scaffolded version of the *L. cuprina* genome (ASM118794v1) and found 10,681 genes (compared to the expected 14,554 genes, data not shown), these results demonstrate that our pipeline for predicting genes is more conservative.

**Table 3:** Male and female *P. regina* gene predictions including the total number of complete genes, the genic structure characteristics (number and length distribution of the intronic and exonic regions), the proportion genes that produced an NCBI result, and the proportion with characterized identifiable protein domains (InterProScan)

	Female	Male
No. of predicted protein-encoding genes	8312	9490
Min length (aa)	66	66
Max length (aa)	14,023	12,632
No. of exons	31,254	33,298
Min length (bases)	3	3
Max length (bases)	14,580	14,589
No. of introns	22,942	23,808
Min length (bases)	42	42
Max length (bases)	55,816	47,389
No. of predicted genes with blast results (NCBI)	7792 (94 %)	8789 (93 %)
InterProScan Statistics		
With InterPro ID’s	6909 (83 %)	7964 (84 %)

In order to compare the genic structures of the predicted genes with other flies, the total counts and average lengths of the exons and introns were compared to recently published genomes of the common housefly *M. domestica* [4], the blow fly *L. cuprina* [64] and the fruit fly *Drosophila melanogaster* (Table 4)*.* The average lengths of gene and intron sequences of *L. cuprina* and *M. domestica* are approximately double the size of *P. regina*’s*.* However, the average lengths of the exons are similar in size among the three species. The cause in the length disparity is likely due to the scaffolded nature of the *Musca* and *Lucilia* genomes, which contain a large number of N’s as placeholders – thus giving rise to seemingly larger introns.

**Table 4:** An overview comparing the genic structure and statistics in *P. regina* (*P.reg*)*, L. cuprina* (*L.cup*) and *M. domestica* (*M.dom*) genome assemblies

	P.reg (Male)	P.reg (Female)	L.cup^a^	M.dom^a^
Genes Count	9490	8312	14,554	15,345
Mean length	6042 bp	6869 bp	12,197 bp	13,553 bp
Exons Count	33,298	31,254	65,493	67,886
Mean length	403 bp	393 bp	432 bp	431 bp
Introns Count	23,808	22,942	–	52,875
Mean length	871 bp	936 bp	2560 bp	3889 bp

^a^Statistics of the genic structure for *L. cuprina* were obtained from Anstead et al in [38], and for *M. domestica* from Scott et al. in

A total of 7792 (94 %) and 8789 (93 %) of the predicted genes in the female and male, respectively, had homology to sequences in GenBank with E-values less than 1*e*-5. Therefore, approximately 6 % of the predicted genes (701 male, 520 female) have no apparent homologs in the arthropod database and could be unique to *P. regina*. Additionally, annotation by InterProScan classified 83.12 and 83.91 % protein domains in the female and male predicted genes, respectively.

The species that were most represented in the BLASTp results for both sexes came from calyptrate flies, specifically the sheep blow fly (*Lucilia cuprina)*, followed distantly by the stable fly (*Stomoxys calcitrans)* and the common house fly (*Musca domestica)* reflecting the phylogenetic relatedness among these species [65]. A total of 5681 (68 %) gene sequences from the female and 5806 (61 %) gene sequences from the male had hits to *L. cuprina* gene sequences while ~7 % of gene sequences from both sexes had top hits from the stable fly and *M. domestica* (Additional file 8: Figure S2).

**Fig. 1:**
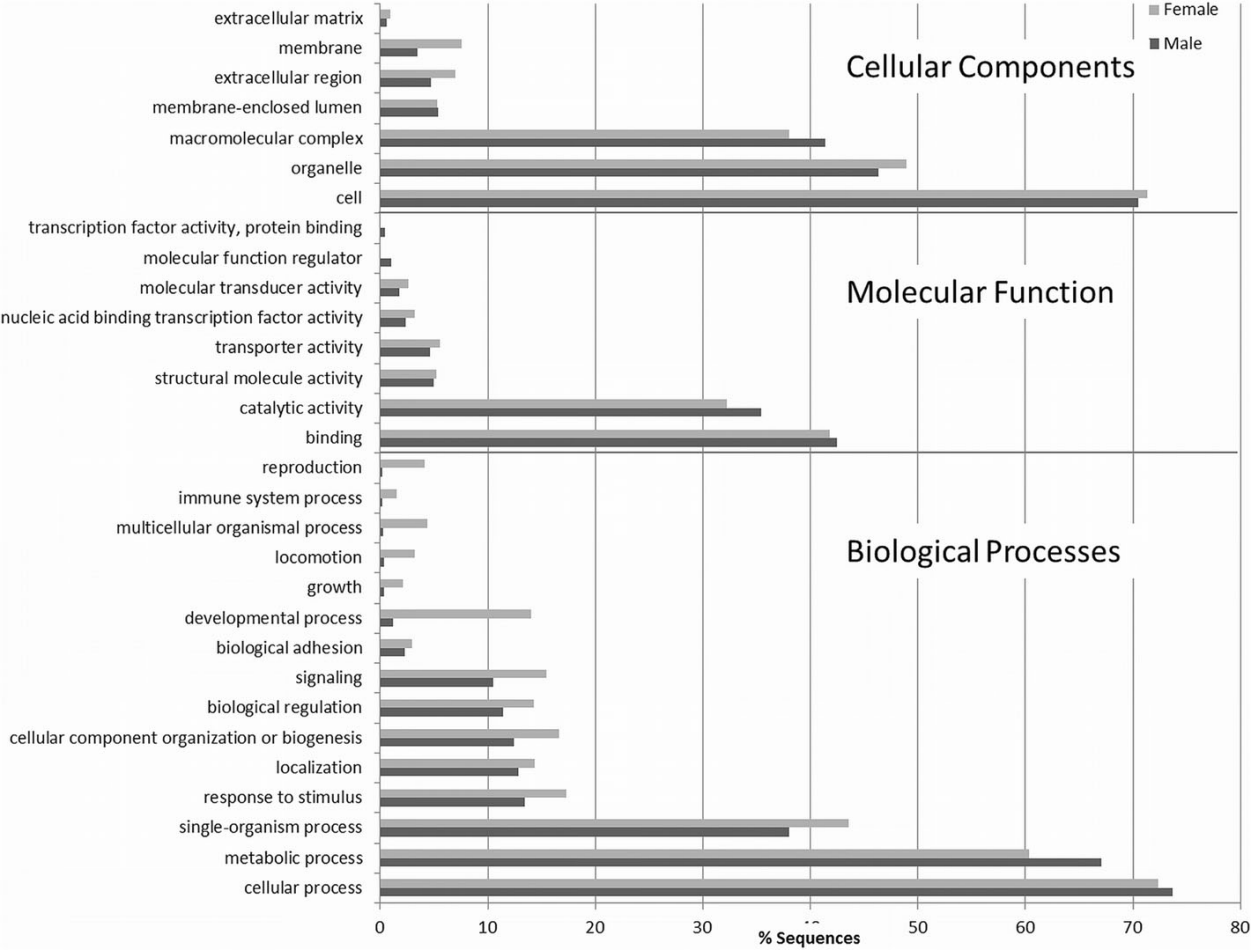
GO term classification of the 3 functional categories (biological processes, molecular function and cellular component) of the predicted genes in the male and female genome assemblies

The two most abundant Biological Process GO categories for both sexes were cellular processes (female 72.4 %, male 73.7 %) and metabolic processes (female 60.4 %, male 67.0 %) (Fig. 1). The GO terms that were associated with Molecular Function were mainly assigned to binding (female 41.8 %, male 42.5 %) and catalytic activity (female 32.3 %, male 35.4 %). While the top GO terms associated with Cellular Component were assigned to cell (female 71.3 %, male 70.5 %) and organelle (female 48.9 %, male 46.4 %) (Additional file 9: Table S7). The overall distribution of genes within GO classifications in *P. regina* was very similar to *M. domestica* and *D. melanogaster* [3] (Additional file 10: Table S8).

**Fig. 2:**
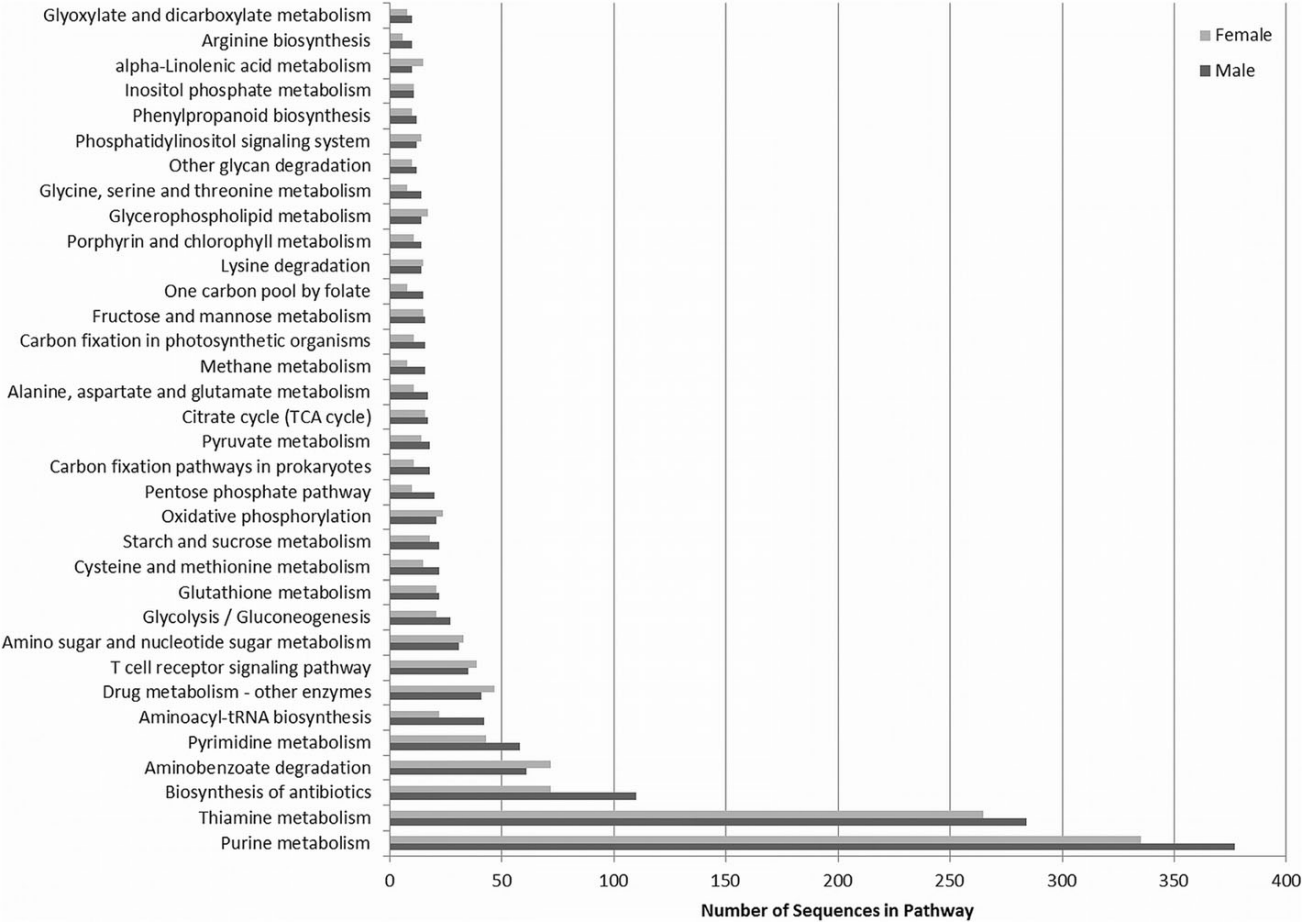
The top 35 KEGG biological pathways of the male and female gene sets extracted from the Blast2GO analysis

Categorization of information from molecular-level interactions extracted from the annotated GO terms was performed by the KEGG component in Blast2GO. A total of 111 and 107 KEGG pathways from the GO-slim Blast2GO analysis were identified for the male and female gene sets, respectively. A visual representation of the pathways with greater than 10 sequences is shown in Fig. 2. The top pathways for both sexes were purine metabolism, thiamine metabolism and biosynthesis of antibiotics. A full list of the KEGG pathways can be found in Additional file 11: Table S9.

### Sex chromosomes

Typically, sex determination is carried out through the heteromorphic XX/XY system where Y-linked male determining genes or the presences of a Y-linked male determining factor are proposed to repress female development causing male sexual differentiation thus promoting the male phenotype [66, 67]. This has been observed in the Mediterranean fruit fly (*Ceratitis capitata*), the olive fruit fly (*Bactrocera oleae*) and the common house fly (*M. domestica*) [68].

Putative sex chromosomes for both the male and female genomes were isolated using the chromosome quotient (CQ) approach [49, 56] and 9134 and 10,721 contigs were identified to putatively belong to the X and Y chromosome, respectively. Adding the sizes of each contig, these result in a putative X chromosome size of ~21.2 Mbp and ~14.5 Mbp for the putative Y chromosome, which approximates measured differences between the male and female flies of ~9 Mbp [43]. A direct comparison with the *Drosophila* X and Y chromosomes yielded 608 (~7 %) female contigs with homology to the X chromosome, and 233 male contigs (~2 %) with homology to the Y chromosome. BLASTn results against the arthropod database (Additional file 12: Table S10) resulted in 47 % (4321 contigs) of the X chromosome contigs and 26 % (2789 contigs) of the Y chromosome contigs identified as having homologous sequences in the database. Most putative Y chromosome contigs did not yield BLAST hits. A reasonable explanation for the limited number of BLAST hits may be the fact that very few model species have characterized and annotated Y chromosomes in the database due to repeat-rich heterochromatic sequences [69, 70].

The majority of the hits from the BLASTn results of the X and Y chromosomes corresponded to repetitive sequences. For example, in the BLAST results from the X chromosome, of the contigs that produced hits, 58 % hit to multiple BAC sequences in *Calliphora vicina* achaete-scute complex, AS-C (accession numbers LN877230-LN877235). Even though these sequences contain the AS-C complex genes, these blast hits are likely hitting to the repetitive regions present in 20–25 % of these BACs [71]. Furthermore, in Drosophilidae*,* the AS-C complex is found in the X chromosome, where *scute* plays an additional role in sex determination acting as an X chromosome signal element [64, 72, 73]. The presence of homologous sequences to the AS-C complex in both the male and female putative sex contigs is an indicator that this complex may also be involved in sex determination pathway in *P. regina.*

#### Unique sex genes

A comparative analysis between the male and female predicted genes showed that 1480 genes were unique to the male and 727 predicted genes were unique to the female. These unique sets of genes are a likely combination of sex-biased genes that drive phenotypic differences leading to sex-specific developmental trajectories [74], and/or be present in either male or female assembly because a complete gene was predicted in one sex and not the other. Approximately 73 % of the male and female unique genes had homology to sequences in NCBI’s Arthropoda NR database. Gene ontology analysis of the unique set of genes for each sex in the biological processes category produced a total of 1589 and 1841 GO terms for the male and female, respectively. Comparing the two sets of GO terms indicated that 517 of the GO terms were unique to the male while 769 were unique to the female. The long lists of the unique GO terms for each sex was summarized by clustering GO terms that belong to the same family and removing redundant terms using the web server REViGO [55].

An example of a clustered category in the male is the category flocculation (Additional file 13: Figure S3A) which include the GO terms sperm motility, energy taxis and positive chemotaxis. The genes functionalized with these GO terms may be specific to the males and involved in sperm chemotaxis where sperm from the male fly is guided by a chemoattractant excreted by the female to fertilize an oocyte [75]. One of the categories clustered in the female (Additional file 13: Figure S3B) is response to xenobiotic stimulus. These are genes expressed during an immune response or during exposure to toxic foreign material (xenobiotic) producing enzymes such as cytochrome P450 and acetyl-CoA synthetases. These female specific genes may be involved in the protection of female flies against the diverse array of pathogens it comes across while laying eggs, or from components of male ejaculates after mating [76]. Similar GO categories connected to immune response were detected to be enriched in female *D. melanogaster* flies as compared to males [77].

### Sex determining genes

In most dipterans investigated thus far, sex determination is regulated by a cascade of genes which exhibit a hierarchical interaction during development where a product of one gene controls the sex-specific splicing of the gene immediately downstream [66, 78]. Some of the key players involved in dipteran sex determination pathway include the genes *sex lethal* (*sxl*), *doublesex* (*dsx*), *transformer* (*tra*), *transformer 2 (tra2)*, *fruitless* (*fru*), *daughterless* (*da*) and *maleless* (*mle*) (Additional file 14: Table S11).

The gene *tra* is one of the main sex determining genes in various insects whose ancestral function is to introduce variation in sex determining mechanisms [79]. We queried both the male and female *P. regina* genomes for homologs using *D. melanogaster* sex determining genes but found none. We then searched for *P. regina tra* and *tra2* using homologs from closely related species - *L. cuprina, Cochliomyia hominivorax, C. macellaria* and *L. sericata*. This search also failed to produce any homologs to *tra* or *tra2* – rather producing homologs to hypothetical *L. cuprina* proteins (FF38_00928 and FF38_09888, respectively). The only homology shared between our putative hits and those in the reference databases are due to the presence of zinc finger domains. For *tra* – *Cochliomyia macellaria* and *C. hominivorax* only share 60.8 % sequence identity. Our putative *tra* gene in *Phormia regina* shares 48.7 % and 52.3 % sequence identity with *Cochliomyia* and *Chrysomya rufifacies,* respectively (data not shown). For *tra2* – a query of our genomes yielded hundreds of hits with e-values less than 1e-52 – suggesting not an assembly error, but rather a common domain is detected. Additional approaches are necessary to more fully annotate these genes in *P. regina*.

**Fig. 3:**
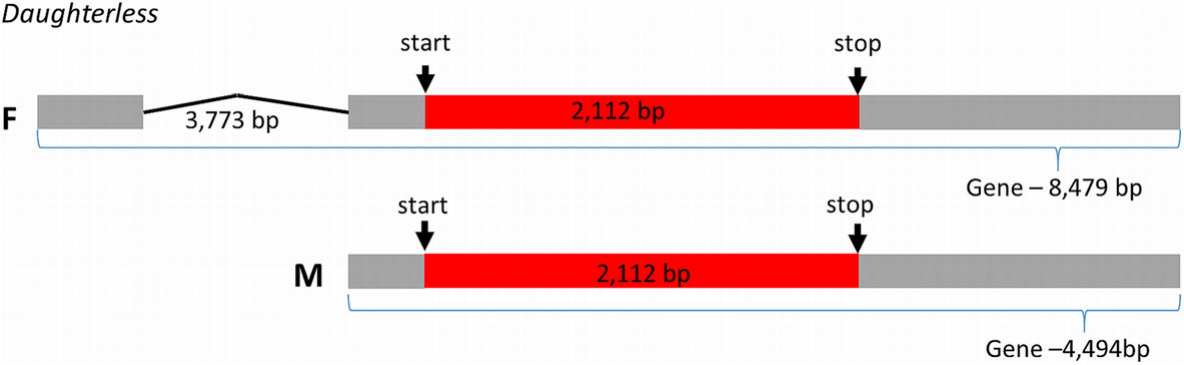
Predicted gene structure of the sex determining gene *daughterless* for the female (F) and male (M) *P. regina.* The red boxes represent the exon, the grey boxes inclusive of the red represent the mRNA, and the black line represent the intron. Image is not drawn to scale

The gene *doublesex* (*dsx*) is another transcription factor that controls the activity of genes involved in sexual differentiation [66, 80]. *Doublesex* is differentially spliced, encoding male and female sex specific *dsx* proteins [66]. Homologous sequences of *dsx* were detected in both sexes with an E-value less than 1*e*-42. The sex determining gene *daughterless* is a member of the basic helix-loop-helix (bHLH) family of DNA binding domains and is a transcription factor [81]. *Da* is essential for neurogenesis, oogenesis and sex determination [82]. We annotated *da* in *P. regina* for both sexes. The length of the predicted *da* gene was determined to be 4494 bp and 8479 bp long in the male and female, respectively, in comparison to the *D. melanogaster* (FBgn0267821) *da* gene (5124 bp) (Fig. 3). The difference between the two appears to be in the 5’ UTR region in which some noncoding gene sequences are predicted in the female, but the male’s 5′ UTR was not completely assembled (the data are missing, Fig. 3).

The coding sequences of *da* for both sexes were nearly identical (99.5 %) and predicted to be 2112 bp long, which is comparable to that of *D. melanogaster* of 2133 bp (J03148.1) and *L. cuprina* 2278 bp (JRES01000453.1 – scaffold966, locus tag FF38_09934). A multiple sequence alignment of the coding sequences demonstrates the high degree of variation with only 87.83 % similarity to *L. cuprina,* and 57.22 % to *D. melanogaster* (Additional file 15: Figure S4). Protein sequences of *da* were also compared between the three species. The length of *da* was 726 amino acid sequences for both sexes in *P. regina* compared to 758 amino acid sequences in *L. cuprina* (KNC31067.1) and 710 amino acid sequences in *D. melanogaster* (P11420). Amino acid sequence alignment shows *L. cuprina* to be 95 % identical to *P. regina,* while *D. melanogaster* is 59 % identical (Additional file 16: Figure S5). A conserved region shared among the three species is the helix-loop-helix domain (Additional file 17: Figure S6).

The *maleless* (*mle*) gene is one of the regulatory genes required for dosage compensation of X-linked genes in the X chromosome of male flies [83]. Annotation of the gene *maleless (mle)* resulted in gene sequences of total length 6375 bp and 6374 bp predicted for the male and female sexes, respectively, approximating the size of *mle* in *D. melanogaster* (6016 bp, JQ663522.1). The length of the protein sequence of *mle* in *P. regina* for both sexes was 1253 amino acids, comparable to *D. melanogaster* (1293 aa, AFI26242) with 72.27 % similarity to *P. regina* (Additional file 18: Figure S7)*.*

### Chemoreceptor genes

A fly’s ability to detect and respond to a chemical signal is integral to its survival. In particular, for blow flies, the adults must be able to detect odors associated with decay immediately following death in order to be among the first insects to show up and lay eggs. Following detection of carrion, an insect must be able to determine the quality of the resource and decide if it is suitable by using a variety of gustatory receptors (GRs), ionotropic receptors (IRs), odorant receptors (ORs), and odorant binding proteins (OBPs). These four gene families are the main chemoreceptors that function in the olfactory and gustatory system of insects [84]. To determine the presence of chemoreceptors in the *P. regina* genomes, we queried *D. melanogaster’s* chemoreceptor genes using tBLASTx. *D. melanogaster* has a predicted total of 68 GRs through alternative splicing, 62 ORs [85], 65 genes encoding IRs [86] and 52 genes encoding OBPs [87]. The tBLASTx results of the predicted chemoreceptors (including alternatively spliced sequences) in *D. melanogaster* resulted in a total of 61 GRs, 40 OBPs, 64 ORs and 63 IR’s with homologous sequences in the female assembled genome. These homologous sequences were detected in 28 contigs (61 GRs), 25 contigs (40 OBPs), 37 contigs (64 ORs) and 41 contigs (63 IRs) (Additional file 1: Table S1).

In addition to the reception of chemical stimulus in olfaction, IR’s have recently been found to be involved in thermosensation, and the circadian cycle [88]. In comparison to ORs, the IR family in insects is relatively conserved suggesting that it is an ancient chemosensory receptor family [89]. *IR25a* is one of the most highly conserved IRs in many species [90]. It acts as a co-receptor with other different odor-sensing IRs. In *D. melanogaster*, it is expressed in sensory neurons in the antennae and also in the proboscis [91]. We compared *IR25a* protein sequences from the female *P. regina* to *L. cuprina, M. domestica, Calliphora stygia, Stomoxys calcitrans, D. melanogaster, C. capitata and B. oleae* via multiple sequence alignment, and subsequent PhyML for tree building, and TreeDyn for tree drawing [92–97]. Overall there was high similarity, with sequence identity ranging from 86 to 97 %. The predicted *P. regina IR25a* sequence was most similar to *L. cuprina* with 97.19 %. The amino acid sequences in regions annotated as putative peptide binding sites were mostly conserved (Additional file 19: Figure S8). The remarkable sequence conservation of *IR25a* implies the presence of a unique and essential evolutionary conserved role of the *IR25a* receptors in various insect species (Additional file 20: Figure S9).

### Antimicrobial genes

Blow flies develop in an environment of decomposing vertebrate tissue that is overrun with bacteria and as such, they not only compete with bacteria for resources but also need protection from infection. Insects in general have a diverse innate immunity pathway that provides protection from various microbes that can inhibit their survival [4, 98, 99]. As a result, they possess different mechanisms that signal the expression of genes activating an antimicrobial defense system to fight bacterial and fungal infections [100]. Some blow fly species have proven useful in wound debridement therapy as they also excrete many potent antimicrobial compounds [31, 33].

Two approaches were used to discover antimicrobial peptide genes: BLASTn using known *D. melanogaster* genes and keyword searching for known antimicrobial peptide sequences against annotation information assigned to the predicted protein sequences (Additional file 21: Table S12). Thirty-six *Drosophila* genes were queried and hits were identified for 25 using BLASTn. Using the BLASTp approach, 115 predicted protein sequences produced BLASTp hits with homology to genes present in four major signaling pathways in insects that are involved with protection from bacterial and fungal infections. These include the Toll, immune deficiency (*Imd*), Janus kinase signal transducer and activator of transcription (JAK/STAT), and JNK pathways [98, 101]. These pathways recognize different types of microbes and induce the transcription of immune-related genes which degrade pathogens or act as signaling molecules [99]. Both male and female had near identical BLASTp results. We therefore selected the female genome for the annotation of the contigs and genes of interest.

The Toll signaling pathway is activated by the presence of gram-positive bacteria or fungi while the *Imd* pathway is mainly activated by gram-negative bacteria. Both pathways lead to the production of antimicrobial peptides to fight pathogens that cause infection [99, 102]. A number of genes involved in the Toll signaling pathway were predicted in the *P. regina* female genome, including *Spaetzle*, *tube*, *toll*, *cactus*, G protein-coupled receptor kinase 2 [103, 104]. This is consistent with the suggestions that the Toll signaling pathway may be involved in the antimicrobial defense system in blow flies as it is in other insects [101, 103, 104]. Furthermore, protein recognition receptors involved in pathogen recognition were identified in *P. regina*. These include peptidoglycan-recognition proteins – *PGRP-LE, PGRP-LC, PGRP-SC2* and gram negative binding proteins – *GNBP1* and *GNBP3*. The NF-kappa β-like gene, *relish*, which is involved in inducing the humoral immune response in *Drosophila* as well as antibacterial and antifungal factors [105] was also identified. Both *relish* and *PGRP-LC* are involved in the *Imd* pathway.

Similar to *M. domestica* and *D. melanogaster*, *P. regina* harbors the four antimicrobial families, attacins, diptericins, cecropins and defensins. We found homology to genes related to antimicrobial humoral responses (*par-1*, *cec*A1 and *tlk* [106, 107]), as well as genes responsible for responses to bacteria (*Gprk*2 and *Relish* [108–110]), all possessing kinase or peptidase activity capable of breaking down bacteria cell walls (Additional file 21: Table S12). These are likely produced and excreted in the salivary glands of the developing larvae as they feed on the decaying tissue. A summary of the top hits from BLASTn results after querying our female genome for the homologous sequences from *D.melanogaster* are listed in Additional file 21: Table S12. The four antimicrobial families were represented by *cecropin A1* (cecropin), *iconoclast* (defensing), *Hephaestus* (diptericin) and *relish* (attacin). These results based on homology to immune-related proteins in other insects, imply that the immune signaling pathway in *P. regina* is similar to other model insects, and may have evolved to work within the harsh sanitary conditions to which *P. regina* is normally exposed.

### Xenobiotic resistance

The enhancement of xenobiotic (foreign compounds) metabolism in insects due to the extensive use of insecticides, has led to the evolution of xenobiotic resistance and tolerance in insects creating a challenge in pest management [111]. The three major groups of genes involved in producing metabolic enzymes to protect insects against plant defense systems (plant allelochemicals) and insecticides are cytochrome P450 monooxygenases, esterases/hydrolases, and glutathione-S-transferases [112, 113]. The presence of these detoxifying enzymes likely helps *P. regina* to withstand high pathogen loads from decaying carrion. Cytochrome P450 genes are the family of enzymes primarily associated with metabolism of xenobiotics and resistance to most insecticides [114, 115]. They are also involved in the catalysis of a range of chemical reactions important for developmental processes.

A total of 41 and 44 predicted genes (Additional file 3: Table S3) were annotated via the *D. melanogaster* database as cytochrome P450 genes from the female and male *P. regina* genes, respectively. This is fewer than in any of the three species we compared; *M. domestica* has 146 P450 genes, 90 in *D. melanogaster*, and 72 in *Glossina morsitans* [116]. However, when we applied our methods to the *Lucilia cuprina* published predicted gene set, we recovered 57 P450 genes (data not shown). *CYP4* and *CYP6* were the predominant P450 families in both male and female draft genomes (Additional file 3: Table S3) where they occupied approximately 36 and 30 % of the total P450 genes respectively (in *L. cuprina*, they occupied 25 and 23 %, respectively). The predominance of *CYP4* and *CYP6* P450 genes was also observed in the genomes of *D. melanogaster* [117] and *M. domestica* where they occupy 50 and >60 % of the total cytochrome P450 genes in their genomes, respectively. The reduction in the number of P450 genes (assumed to be closer to 100 in insect genomes) is likely due to the stringent gene prediction criteria used in our fragmented genomes (only complete genes predicted).

An increase in the expression or activity of the metabolic enzymes that belong to the esterases and hydrolases family has also been linked to insecticide resistance [118] and correlated with resistance to two major insecticide classes: pyrethroids and organophosphates [113]. This is mainly due to the presence of ester bonds in most insecticides which are hydrolyzed by the esterase [119]. A total of 103 and 131 genes with hydrolase/esterase activities were predicted in the female and male draft genomes, respectively. The common ones in both sexes included phosphodiesterase, thioesterase, carboxylesterase and phosphatase (Additional file 5: Table S5).

Glutathione S-transferases (GST) are multifunctional enzymes that are not only involved in detoxification of xenobiotic compounds, but also in other physiological processes in insects including intracellular transport and hormone biosynthesis [120]. In the detoxification process, they function by metabolizing insecticides producing water-soluble metabolites that are easily excreted [121, 122]. A total of 9 and 11 GST genes were predicted in the female and male *P. regina* genomes, respectively (Additional file 4: Table S4). Unfortunately, insecticide resistance is a growing problem [123] in many insects, including mosquitos [124–126] and blow flies [127–129] has in some cases been attributed to glutathione S-transferase activity [120, 123, 130–132].

### Repetitive elements

Repeat identification in the male and female genomes via homology-based searches identified close to 38 and 46 Mbp of repetitive DNA accounting for 7.3 and 8.7 % of the male and female assemblies (Additional file 22: Table S13). Approximately 10.5 (male) and 18 Mbp (female) of repetitive sequences were transposable elements, and the remaining were low complexity sequences including mini- and microsatellites. Several major transposable elements super-families were identified in the *P. regina* genome, but the vast majority (>70 %) of elements belonged to 5 families or super families: Jockeys, LOAs, Gypsys, Tc-Mariners, and Helitrons. DNA transposons and retrotransposons were present in roughly equal proportions. Low complexity sequences were the dominant type of repetitive sequence making up ~66 % of the male and female genome assemblies.

Dipteran species show significant variability in SINE content but SINE elements appear to be missing from the *P. regina* genome entirely. For example, SINEs are absent in *Drosophila* [133] but present in high copy numbers in other dipterans [5]. Only 39 SINE insertions were identified in *P. regina*, most of which are distantly related, or highly mutated version of SINE-3_QC and SINE-4_QC from *Culex quinquefasciatus*, the southern house mosquito. In *C. quinquefasciatus*, SINE-3_QC and SINE-4_QC are present more than ten thousand times [134].

**Fig. 4:**
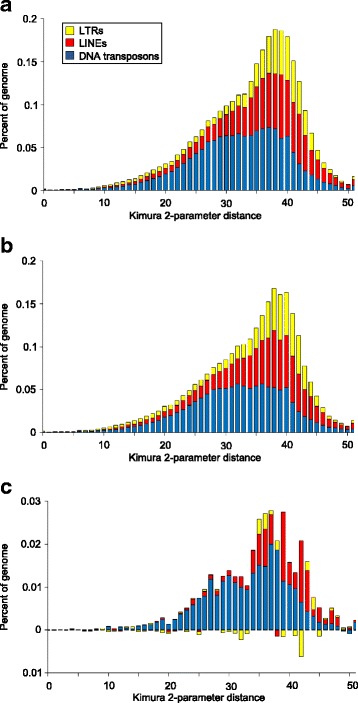
Transposable element accumulation in the female (**a**) and male (**b**) *Phormia regina* genome assemblies. Kimura 2-parameter distances were calculated between transposable element insertions in the genome and the homologous element in the dipteran Repbase library. Larger divergences indicate elements with larger mutation loads, and by extension, were deposited in the genome in the more distant past. Less than 40 SINE elements are present in either the female or male assemblies and are not shown here. Transposable elements are slightly more abundant in the female genome assembly. The accumulation of female specific repeats (**c**) follows that of the whole genome in general

Based on the female (Fig. 4a) and male (Fig. 4b) accumulation profiles, it appears that transposable elements in *P. regina* tend to be old, with little accumulation in the recent past. In general, transposable element insertions for each family tend to be more numerous in the female genome assembly. Transposable elements present in the female assembly, but absent in the male assembly follow a similar accumulation profile to the genomes as a whole (Fig. 4c) ruling out temporally biased accumulation in either sex. Class II transposons, LINEs, and LTRs accumulated at similar times given that the majority of elements in each group are between 37 and 45 % diverged from their putative ancestral partner. DNA transposons have been accumulating for a slightly longer period than LINEs and LTRs with most element divergences ranging from 23 to 41 %. In all, >97 % of all elements are >10 % divergent from their respective consensus elements. This implies that the minimal transposable elements accumulation has occurred in the recent past, or that newly inserted transposable elements are being actively removed from the genome [135]. However, with high divergences between potentially novel transposable elements in *P. regina* and transposable elements in RepBase is possible that lineage-specific SINEs are present but unidentifiable using homology based searches [136] and a full TE curation of the genome is necessary.

Close to 1.7 Mb of the X chromosome (8.1 % of the total X chromosomes) was derived from repeats, compared to ~390 Kb of the Y chromosome (2.7 % of the total Y chromosome). For both chromosomes, more than half the repetitive sequences are in the form of simple repeats (60 and 55 % for the X and Y chromosomes, respectively). The increased amount of repeats on the X chromosome is likely due to its larger size, however, even though the X chromosome is proportionally more repetitive than the Y, it is not large enough to be significant.

## Conclusions

Although all impacts are impossible to foresee, we anticipate four important fields will benefit from this data: (1) insecticide resistance and/or sensitivity; (2) adaptive evolution in a rapidly evolving clade (Calliphoridae); (3) sex chromosome evolution; and (4) genotype-phenotype correlations of development rate variation.

### Insecticide resistance

There are four main calliphorids that are serious agricultural pests causing myiasis (infestation by fly larvae): *Chrysomya bezziana* (Old World screwworm), *Cochliomyia hominivorax* (New World screwworm), *Lucilia sericata* (green bottle fly) and *L. cuprina* (sheep blow fly) [14, 137, 138], although other species have been implicated in secondary myiasis, including *P. regina* ([139–143]. Two of these, *Ch. bezziana* and *Co. hominivorax* are obligate parasites, while the remaining two *Lucilia* species are either obligate, or facultative ectoparasites, depending on where they reside [144]. The genome of the obligate ectoparasite *L. cuprina* (in Australia/New Zealand, this species does not appear to ever develop on carrion) was recently sequenced in an effort to determine how insecticide resistance has persisted and to develop novel pest management targets that would not harm beneficial insects. In order to better understand these processes and assess target efficacy, it is important to understand how these genes are structured in other closely related flies. The *P. regina* reference genome presented here will provide the opportunity to extract candidate genes, determine the structure-function relationship, and produce new ligands with the potential to slow or eradicate these pest species.

### Adaptive evolution

Calliphoridae includes approximately 1500 different species and accounts for ~8 % of calyptrate species diversity [8]. Many lineages within Calliphoridae have evolved specialized adaptations. As the above example demonstrates, ectoparasitism has evolved at least twice within Calliphoridae, perhaps in response to selective pressures of the usually ephemeral carrion resource – if the insect can arrive at a resource before it becomes available to all its competitors, its genes have a greater probability of being maintained. *P. regina* does not have these features or life-strategies, suggesting the retention of the ancestral life history, and can thus serve as a robust reference genome from which more derived features can be understood.

### Sex chromosome evolution

Many calyptrate flies utilize XY sex determination. In many other species sharing this system, the X chromosome is relatively stable, and the Y chromosome has experienced loss of function due to the absence of recombination over time and the presence of repetitive DNA. Therefore, the structure of the Y chromosome represents its approximate evolutionary age. With individual sex genomes, and putative chromosomes sequenced and assembled herein, we can compare sex chromosome structure within the Calliphoridae, where some species have vastly different sex chromosome sizes (i.e. *Lucilia cuprina* male and female flies differ by nearly 100 Mbp, [43]), or within the monogenic fly *Chrysomya rufifacies* with no difference in genome sizes of the male and female flies [43]. Additionally, this reference genome will be useful in studying sex determining pathways in Calliphoridae, which could be useful as a mechanism to target for pest control [145].

### Development rate variation

As many of these flies are primary colonizers of vertebrate carrion [17], they have forensic uses in cases of decomposition when the time of death cannot be determined using traditional physiological approaches. The collected larvae serve as a clock to the minimum time since the individual died, as they will only colonize a body following death (excluding species known to cause myiasis). Then, the age of the larvae is estimated for a minimum postmortem interval [146, 147]. The age, however, is estimated from a reference data set in which laboratory conditions permit multiple temperatures and conditions [148–150]. These models of development assume little to no population-level variation even though variation has been clearly demonstrated [151–153]. Thus, it is important to understand the fundamental developmental processes in blow flies if they are to be used for postmortem interval estimates, and to be capable to predicting the possible variation based on genotypes of the individual larvae collected. Once again, the availability of a reference genome, differentiating male and female genomes [154], will be invaluable to understanding the molecular basis of development, and its associated variation.

This first draft of the *P. regina* genome represents a critical step in calliphorid genomics. The accessibility of the *P. regina* genome will quicken the pace of the exploration and comparisons in the evolutionary relationships and developmental analysis among blow fly species and also with other dipteran species. In time, these findings could have significant agricultural, medical and forensics fields.

## Additional files

Additional file 1: Table S1. tBLASTx results of contigs with *D. melanogaster’s* homologus gene sequences of gustatory receptors, odorant binding proteins, odorant receptors and ionotropic receptors. (XLSX 22 kb)
Additional file 2: Table S2. BLASTn (27 hits) and BLASTp (115 hits) results for antimicrobial peptide queries using *D. melanogaster*’s homologous gene and protein sequences as queries. (XLSX 19 kb)
Additional file 3: Table S3. A list showing the distribution of cytochrome P450 monooxygenases families in the male and female *P. regina* genomes. (XLSX 18 kb)
Additional file 4: Table S4. A list showing the distribution of glutathione S-transferases in the male and female *P. regina* genomes. (XLSX 9 kb)
Additional file 5: Table S5. A list of metabolic enzymes that belong to the esterases and hydrolases family that are predicted to be linked with insecticide resistance. (XLSX 22 kb)
Additional file 6: Figure S1. The mitochondrial DNA (mtDNA) genome of *Phormia regina*, annotated using the mtDNA genome of *Cochliomyia hominivorax* mtDNA genome. The AT-rich region is colored in purple, tRNA and rRNA genes in red, and protein coding genes are in yellow. (JPG 102 kb)
Additional file 7: Table S6. CEGMA Completeness report of the female and male *P. regina* genomes. Number of proteins equals the number of proteins found from the 248 ultra-conserved CEGs present in the genome with % completeness representing the percentage. (DOC 48 kb)
Additional file 8: Figure S2. Top-hit species distribution from Blast2GO for the male and female *P. regina*. The top hit species is the blow fly *L. cuprina*. The species listed are those with >25 hits. Those with less than the threshold are summed and grouped in the ‘Other’ category. (JPG 551 kb)
Additional file 9: Table S7. Comparative gene ontology terms of the male and the female *Phormia regina* predicted genes. (DOC 69 kb)
Additional file 10: Table S8. A comparative gene ontology showing the ranking of different functional categories in each species among *P. regina* (both sexes), *M. domestica* and *D. melanogaster* with the rank of 1 indicating the most abundant GO term. (DOC 62 kb)
Additional file 11: Table S9. List of biological KEGG pathways extracted from the annotations of the male and female predicted protein-encoding genes. Each tab represents biological KEGG pathways extracted from the male and female genomes. (XLSX 66 kb)
Additional file 12: Table S10. BLASTn results of the putative sex chromosome contigs (chromosome X and Y) queried against the arthropoda database in GenBank. (XLSX 729 kb)
Additional file 13: Figure S3. A visual representation showing a summary of GO terms categorized in the biological processes that are unique to the male (A) and female (B). (JPG 1742 kb)
Additional file 14: Table S11. Top hits of sex determining genes homologous to *D. melanogaster* in the male and female *P. regina* assembled genomes. (DOC 34 kb)
Additional file 15: Figure S4. Multiple sequence alignment of the coding sequences of the sex determining gene *daughterless* of male and female *P. regina, L. cuprina* (scaffold 966) and *D. melanogaster* (J03148)*.* Sequence similarities between the male and female *P. regina* is 99.95 %, between *P. regina* (F) and *L. cuprina* is 87.83 %, and between *P. regina* (F) and *D. melanogaster* is 57.22 %. (JPG 1974 kb)
Additional file 16: Figure S5. Multiple sequence alignment of protein sequences of the sex determining gene *daughterless* of *P. regina, L. cuprina* (KNC31067) and *D. melanogaster* (P11420)*.* Sequence similarity of *P. regina* to *L. cuprina* is 95 % and to *D. melanogaster* is 59 %. (JPG 1589 kb)
Additional file 17: Figure S6. A section of a multiple sequence alignment of the sex determining gene, *daughterless*, of the conserved region of 60 amino acid sequences of the helix-loop-helix domain of the da protein. (JPG 593 kb)
Additional file 18: Figure S7. Multiple sequence alignment of protein sequences of the sex determining gene *maleless* of *P. regina* and *D. melanogaster* (P24785). Sequence similarities between the male and female *P. regina* is 100 %, and the similarity to *D. melanogaster* is 72.27 %. (JPG 1574 kb)
Additional file 19: Figure S8. Multiple amino acid sequence alignment of the predicted ionotropic receptor IR25a in *Phormia regina* (g4045.t1), *Lucilia cuprina* (KNC28739, sequence similarity 97.19 %)*, Stomoxys calcitrans* (XP_013104244, sequence similarity 94.76 %), *Bactrocera oleae* (XP_014086336, sequence similarity 88.12 %)*, Ceratitis capitata* (XP_004530416, sequence similarity 87.85 %). Also included are protein sequences generated from sequenced RNA from *Calliphora stygia* (AID61273, sequence similarity 97.06 %)*, Musca domestica* (NP_001273813, 93.36 %)*,* and *Drosophila melanogaster* (NP_001260049, sequence similarity 86.08 %). The *P. regina* amino acid sequence is incomplete at the amino terminus, however, the conservation in this protein is demonstrated by the similarities between the wide taxonomic groups represented here. (JPG 1574 kb)
Additional file 20: Figure S9. Maximum likelihood phylogenetic tree of the amino acid sequences of IR25a based on alignment generated for Additional file 19: Figure S8. Scale bar represents evolutionary distance (number of amino acid substitutions). (JPG 148 kb)
Additional file 21: Table S12. A summary of the top BLASTn results of antimicrobial related genes homologous to *Drosophila*. The four antimicrobial families are represented by *cecropin* A1 (cecropin), *iconoclast* (defensin), *hephaestus* (diptericin) and *relish* (attacin). (DOC 37 kb)
Additional file 22: Table S13. A summary of copy number, number of bases, and percent of the genome derived from repetitive sequences in the female and male *Phormia regina* genome. Repeats were identified based on homology to known dipteran repetitive sequences in RepBase. (DOC 72 kb)

## Abbreviations

aa: Amino acid
AS-C: Achaete-scute complex
BAC: Bacterial artificial chromosome
bHLH: Basic helix-loop-helix
bp: Base pair
CEGMA: Core eukaryotic genes mapping approach
CoA: Coenzyme A
CQ: Chromosome quotient
*D. melanogaster*: *Drosophila melanogaster*
*da*: *daughterless*
*dsx*: *doublesex*
*fru*: *fruitless*
Gb: Gigabase
GNBP: Gram negative binding proteins
GO: Gene ontology
GRs: Gustatory receptors
GSTs: Glutathione S-transferases
*Imd*: Immune deficiency
IRs: Ionotropic receptors
JAK/STAT: Janus kinase signal transducer and activator of transcription
KEGG: Kyoto Encyclopedia of Genes and Genomes
*L. cuprina*: *Lucilia cuprina*
LINEs: Long interspersed nuclear elements
LTRs: Long terminal repeats
Mb: Megabases
Mbp: Million base pair
*mle*: *Maleless*
MRSA: Methicillin resistant *Staphylococcus aureus*
OBPs: Odorant binding proteins
ORs: Odorant receptors
*P. regina*: *Phormia regina*
PGRP: Peptidoglycan-recognition proteins
REViGO: Reduce and visualize gene ontology
SINEs: Short interspersed nuclear elements
*sxl*: *Sex lethal*
*tra*: *Transformer*
*tra2*: *Transformer2*
UTR: Untranslated region
