# Supplementary figures and images for: Genome sequence of Phormia regina Meigen (Diptera: Calliphoridae): implications for medical, veterinary and forensic research
Source: BMC Genomics. 2016 Oct 28;17:842. doi: 10.1186/s12864-016-3187-z (PMC5084420; doi:10.1186/s12864-016-3187-z)

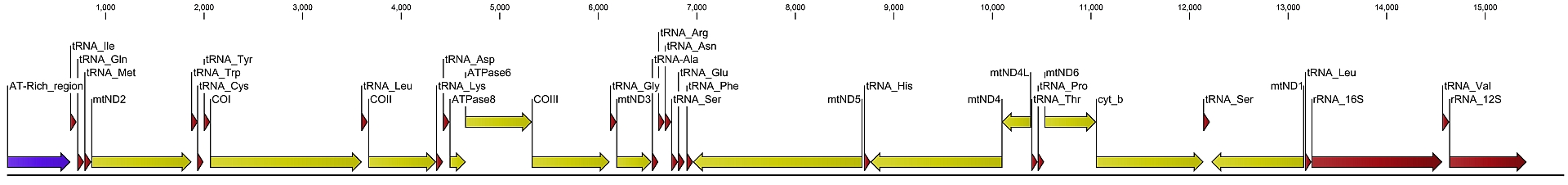

Supplement: Additional file 6: Figure S1. — The mitochondrial DNA (mtDNA) genome of Phormia regina, annotated using the mtDNA genome of Cochliomyia hominivorax mtDNA genome. The AT-rich region is colored in purple, tRNA and rRNA genes in red, and protein coding genes are in yellow. (JPG 102 kb) [file 12864_2016_3187_MOESM6_ESM.jpg]

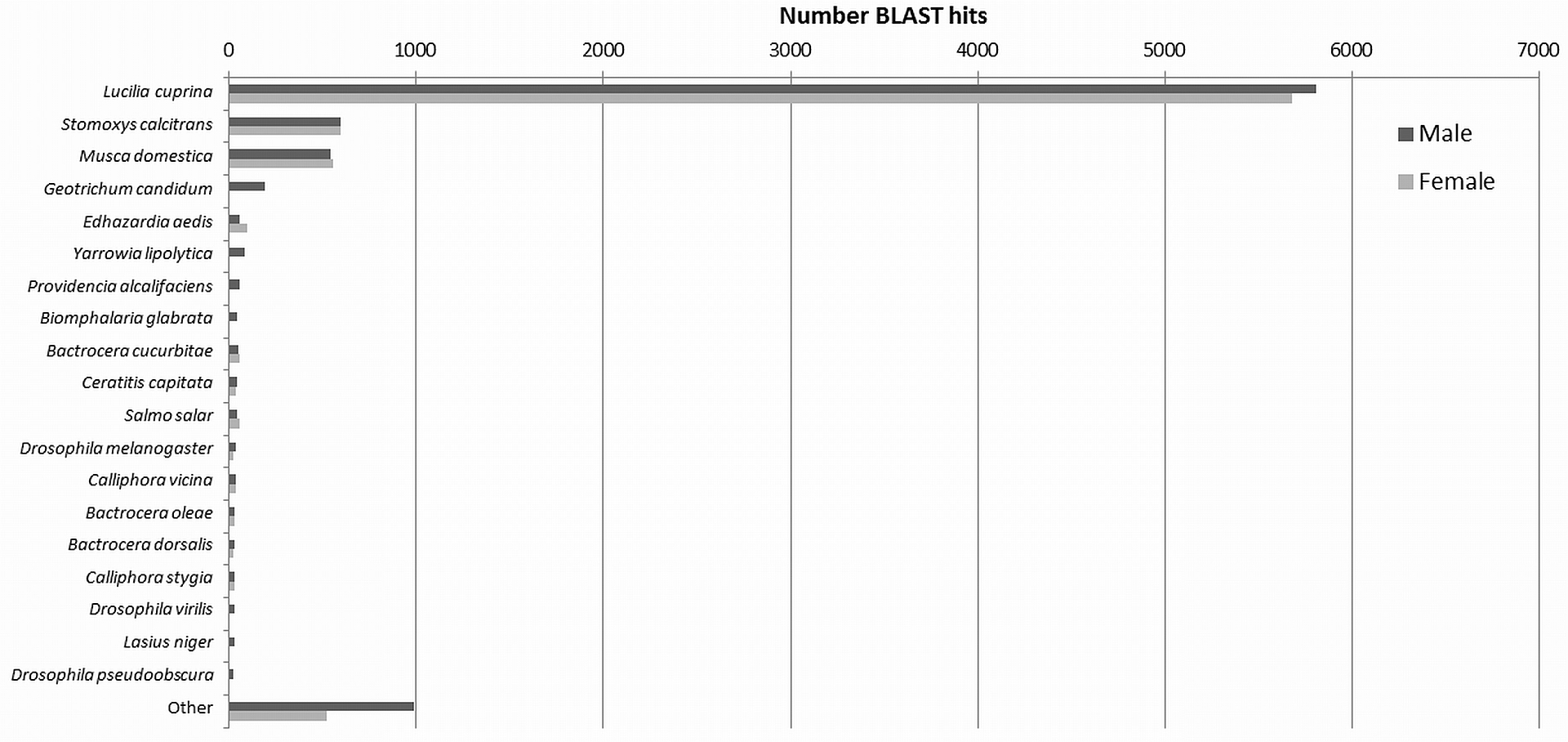

Supplement: Additional file 8: Figure S2. — Top-hit species distribution from Blast2GO for the male and female P. regina. The top hit species is the blow fly L. cuprina. The species listed are those with >25 hits. Those with less than the threshold are summed and grouped in the ‘Other’ category. (JPG 551 kb) [file 12864_2016_3187_MOESM8_ESM.jpg]

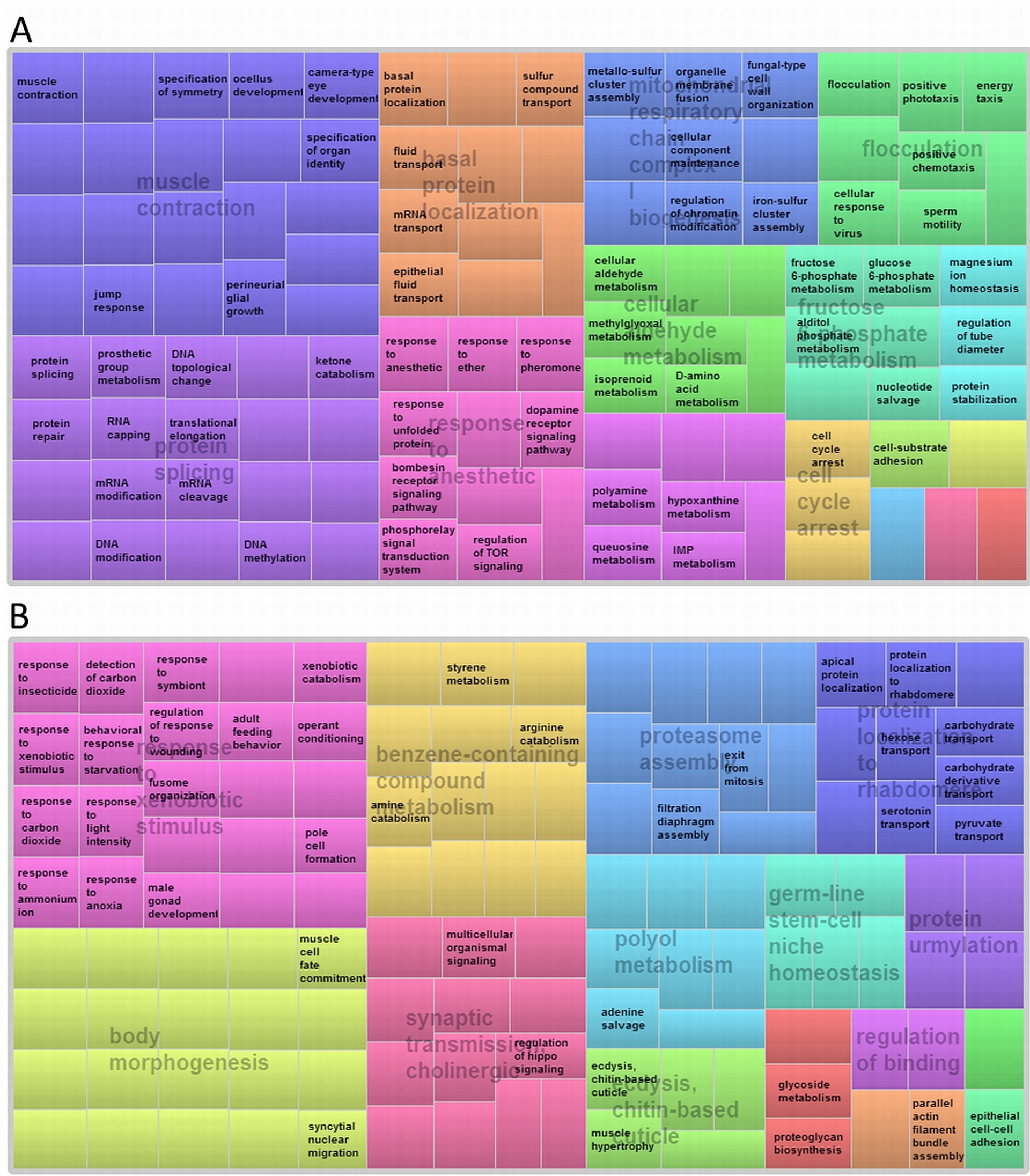

Supplement: Additional file 13: Figure S3. — A visual representation showing a summary of GO terms categorized in the biological processes that are unique to the male (A) and female (B). (JPG 1742 kb) [file 12864_2016_3187_MOESM13_ESM.jpg]

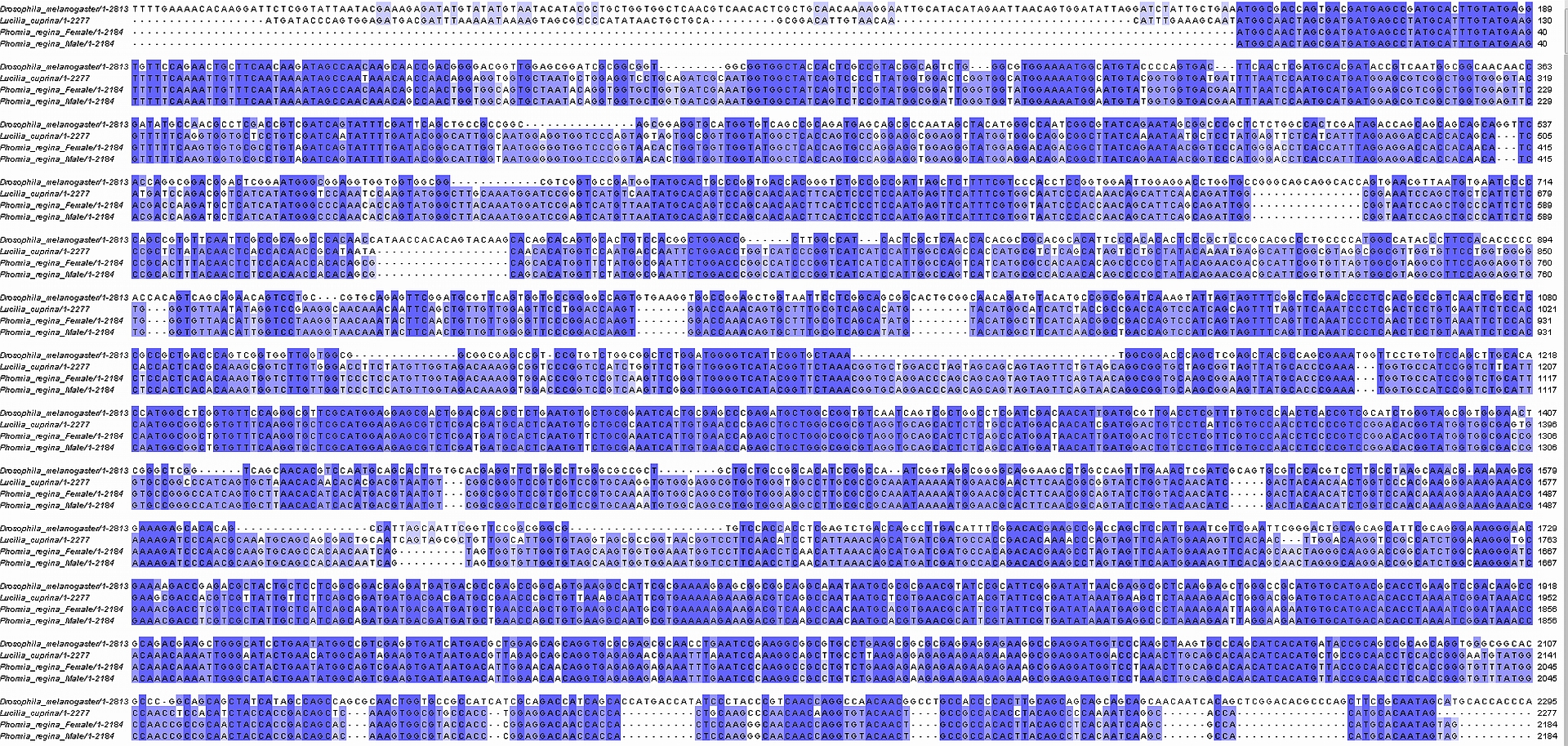

Supplement: Additional file 15: Figure S4. — Multiple sequence alignment of the coding sequences of the sex determining gene daughterless of male and female P. regina, L. cuprina (scaffold 966) and D. melanogaster (J03148). Sequence similarities between the male and female P. regina is 99.95 %, between P. regina (F) and L. cuprina is 87.83 %, and between P. regina (F) and D. melanogaster is 57.22 %. (JPG 1974 kb) [file 12864_2016_3187_MOESM15_ESM.jpg]

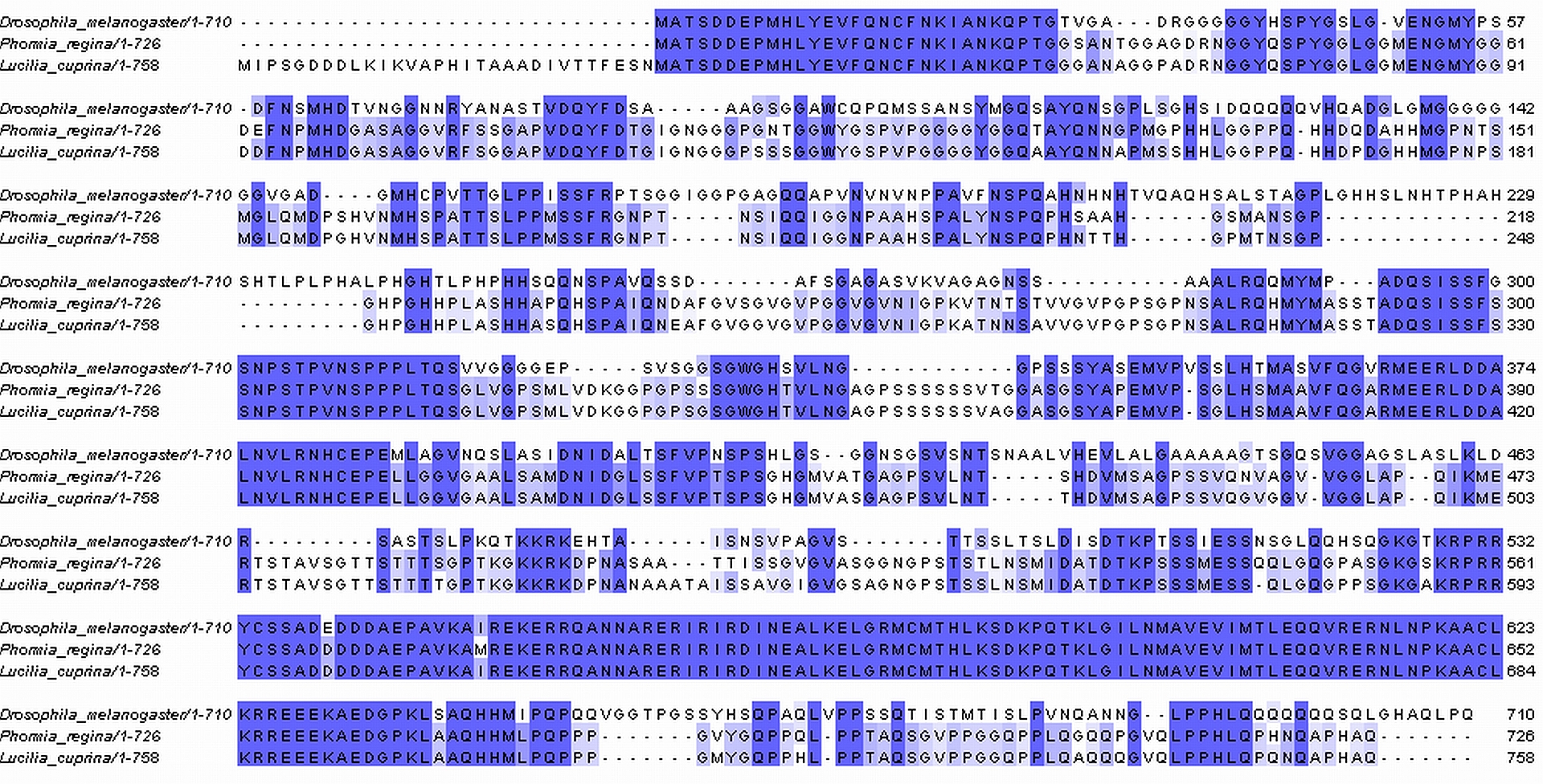

Supplement: Additional file 16: Figure S5. — Multiple sequence alignment of protein sequences of the sex determining gene daughterless of P. regina, L. cuprina (KNC31067) and D. melanogaster (P11420). Sequence similarity of P. regina to L. cuprina is 95 % and to D. melanogaster is 59 %. (JPG 1589 kb) [file 12864_2016_3187_MOESM16_ESM.jpg]

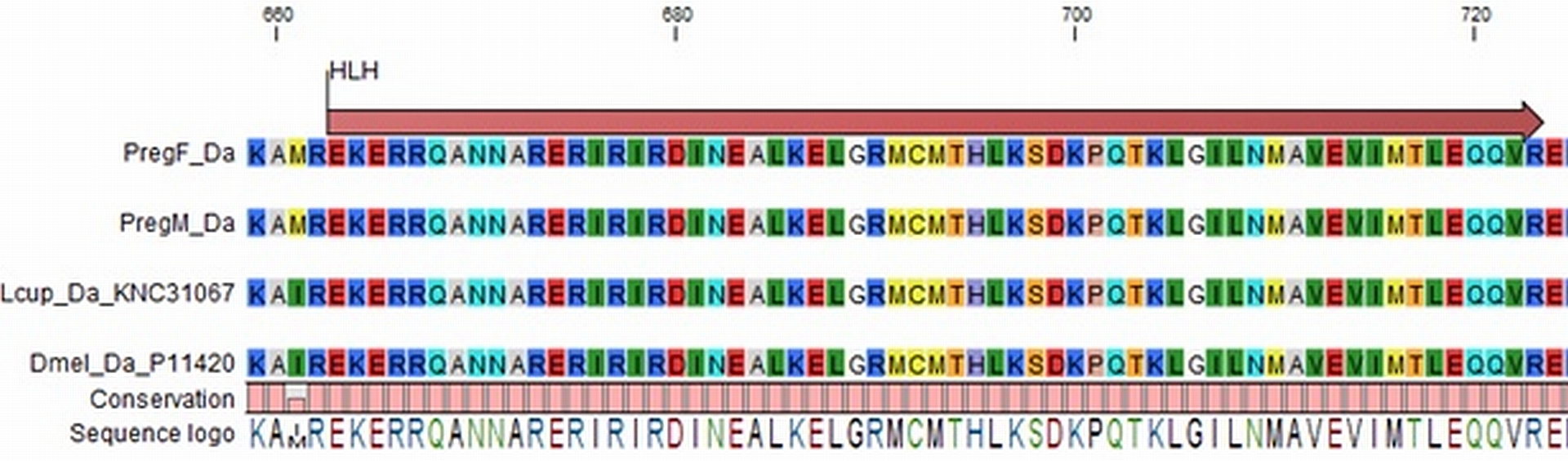

Supplement: Additional file 17: Figure S6. — A section of a multiple sequence alignment of the sex determining gene, daughterless, of the conserved region of 60 amino acid sequences of the helix-loop-helix domain of the da protein. (JPG 593 kb) [file 12864_2016_3187_MOESM17_ESM.jpg]

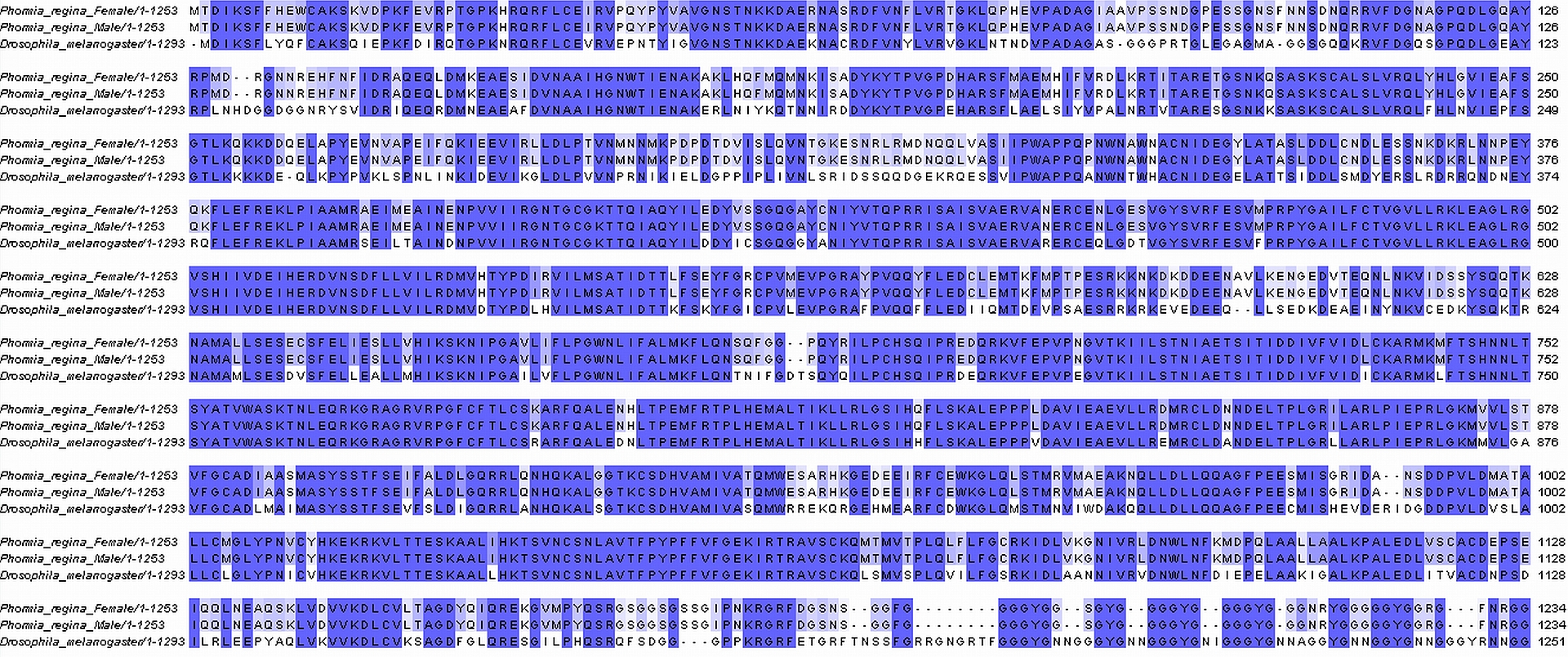

Supplement: Additional file 18: Figure S7. — Multiple sequence alignment of protein sequences of the sex determining gene maleless of P. regina and D. melanogaster (P24785). Sequence similarities between the male and female P. regina is 100 %, and the similarity to D. melanogaster is 72.27 %. (JPG 1574 kb) [file 12864_2016_3187_MOESM18_ESM.jpg]

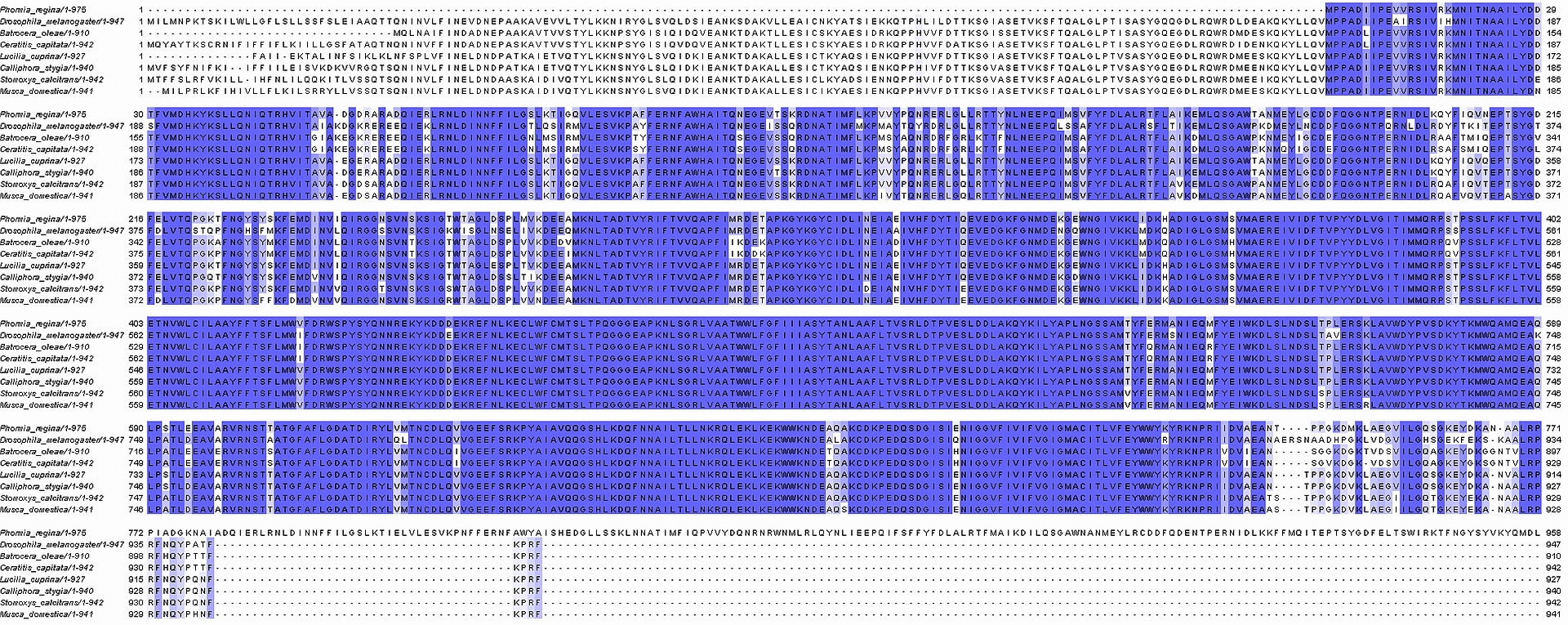

Supplement: Additional file 19: Figure S8. — Multiple amino acid sequence alignment of the predicted ionotropic receptor IR25a in Phormia regina (g4045.t1), Lucilia cuprina (KNC28739, sequence similarity 97.19 %), Stomoxys calcitrans (XP_013104244, sequence similarity 94.76 %), Bactrocera oleae (XP_014086336, sequence similarity 88.12 %), Ceratitis capitata (XP_004530416, sequence similarity 87.85 %). Also included are protein sequences generated from sequenced RNA from Calliphora stygia (AID61273, sequence similarity 97.06 %), Musca domestica (NP_001273813, 93.36 %), and Drosophila melanogaster (NP_001260049, sequence similarity 86.08 %). The P. regina amino acid sequence is incomplete at the amino terminus, however, the conservation in this protein is demonstrated by the similarities between the wide taxonomic groups represented here. (JPG 1574 kb) [file 12864_2016_3187_MOESM19_ESM.jpg]

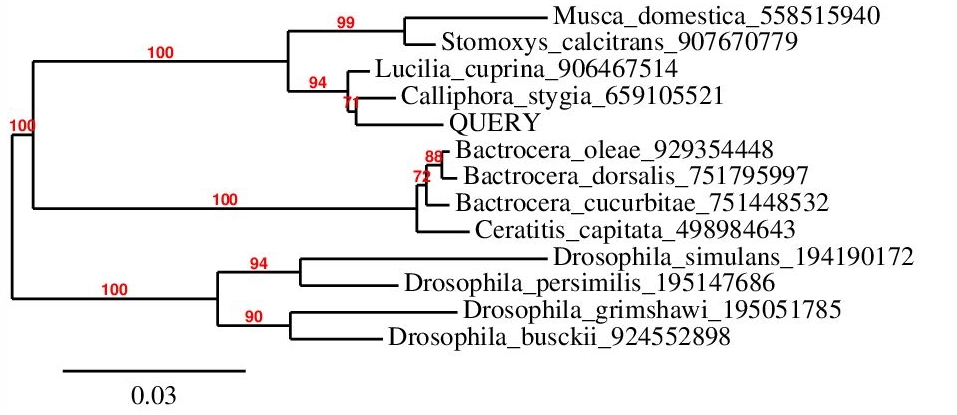

Supplement: Additional file 20: Figure S9. — Maximum likelihood phylogenetic tree of the amino acid sequences of IR25a based on alignment generated for Additional file 19: Figure S8. Scale bar represents evolutionary distance (number of amino acid substitutions). (JPG 148 kb) [file 12864_2016_3187_MOESM20_ESM.jpg]
