# Supplementary material for: Genome sequence of Phormia regina Meigen (Diptera: Calliphoridae): implications for medical, veterinary and forensic research
Source: BMC Genomics. 2016 Oct 28;17:842. doi: 10.1186/s12864-016-3187-z (PMC5084420; doi:10.1186/s12864-016-3187-z)
Supplement: Additional file 7: Table S6. — CEGMA Completeness report of the female and male P. regina genomes. Number of proteins equals the number of proteins found from the 248 ultra-conserved CEGs present in the genome with % completeness representing the percentage. (DOC 48 kb) [file 12864_2016_3187_MOESM7_ESM.doc]

Table S6. CEGMA Completeness report of the female and male *P. regina* genomes. Number of proteins equals the number of proteins found from the 248 ultra-conserved CEGs present in the genome with % completeness representing the percentage.

|  | **# Proteins** | **% Completeness** | **Total # CEGs** | **Average # Orthologs per CEG** | **% CEGs with >1 Orthologs** |
| --- | --- | --- | --- | --- | --- |
| **Female** | | | | | |
| **Complete** | 233 | 93.95 | 338 | 1.45 | 36.05 |
| **Group 1** | 61 | 92.42 | 85 | 1.39 | 34.43 |
| **Group 2** | 53 | 94.64 | 72 | 1.36 | 28.30 |
| **Group 3** | 58 | 95.08 | 85 | 1.47 | 41.38 |
| **Group 4** | 61 | 93.85 | 96 | 1.57 | 39.34 |
| **Partial** | 246 | 99.19 | 407 | 1.65 | 45.93 |
| **Group 1** | 66 | 100.00 | 96 | 1.45 | 36.36 |
| **Group 2** | 55 | 98.21 | 81 | 1.47 | 34.55 |
| **Group 3** | 60 | 98.36 | 106 | 1.77 | 55.00 |
| **Group 4** | 65 | 100.00 | 124 | 1.91 | 56.92 |
| **Male** | | | | | |
| **Complete** | 240 | 96.77 | 454 | 1.89 | 56.25 |
| **Group 1** | 62 | 93.94 | 100 | 1.61 | 37.10 |
| **Group 2** | 55 | 98.21 | 100 | 1.82 | 50.91 |
| **Group 3** | 59 | 96.72 | 115 | 1.95 | 61.02 |
| **Group 4** | 64 | 98.46 | 139 | 2.17 | 75.00 |
| **Partial** | 247 | 99.60 | 627 | 2.54 | 79.76 |
| **Group 1** | 66 | 100.00 | 138 | 2.09 | 65.15 |
| **Group 2** | 56 | 100.00 | 137 | 2.45 | 78.587 |
| **Group 3** | 60 | 98.36 | 161 | 2.68 | 83.33 |
| **Group 4** | 65 | 100.00 | 191 | 2.94 | 92.31 |
