# Supplementary material for: Genome sequence of Phormia regina Meigen (Diptera: Calliphoridae): implications for medical, veterinary and forensic research
Source: BMC Genomics. 2016 Oct 28;17:842. doi: 10.1186/s12864-016-3187-z (PMC5084420; doi:10.1186/s12864-016-3187-z)
Supplement: Additional file 9: Table S7. — Comparative gene ontology terms of the male and the female Phormia regina predicted genes. (DOC 69 kb) [file 12864_2016_3187_MOESM9_ESM.doc]

Table S7: Comparative gene ontology terms of the male and the female *Phormia regina* predicted genes.

|  | Term | Male | | Female | |
| --- | --- | --- | --- | --- | --- |
|  |  | %Sequences | Rank | %Sequences | Rank |
| **Biological Processes** | cellular process | 73.65 | 1 | 72.36 | 1 |
|  | metabolic process | 67.03 | 2 | 60.38 | 2 |
|  | single-organism process | 37.99 | 3 | 43.58 | 3 |
|  | response to stimulus | 13.4 | 4 | 17.31 | 4 |
|  | localization | 12.84 | 5 | 14.32 | 7 |
|  | cellular component organization or biogenesis | 12.39 | 6 | 16.66 | 5 |
|  | biological regulation | 11.39 | 7 | 14.25 | 8 |
|  | signaling | 10.47 | 8 | 15.49 | 6 |
|  | biological adhesion | 2.25 | 9 | 2.96 | 13 |
|  | developmental process | 1.16 | 10 | 14.04 | 9 |
|  | growth | 0.32 | 11 | 2.07 | 14 |
|  | locomotion | 0.32 | 12 | 3.2 | 12 |
|  | multicellular organismal process | 0.24 | 13 | 4.37 | 10 |
|  | immune system process | 0.16 | 14 | 1.51 | 15 |
|  | reproduction | 0.16 | 15 | 4.1 | 11 |
|  | multi-organism process | 0.04 | 16 | 0.07 | 16 |
| **Molecular Function** | binding | 42.48 | 1 | 41.79 | 1 |
|  | catalytic activity | 35.42 | 2 | 32.27 | 2 |
|  | structural molecule activity | 4.96 | 3 | 5.22 | 4 |
|  | transporter activity | 4.61 | 4 | 5.56 | 3 |
|  | nucleic acid binding transcription factor activity | 2.36 | 5 | 3.22 | 5 |
|  | molecular transducer activity | 1.8 | 6 | 2.61 | 6 |
|  | molecular function regulator | 1.05 | 7 | 0 | 0 |
|  | transcription factor activity, protein binding | 0.42 | 8 | 0 | 0 |
|  | enzyme regulator activity | 0 | 0 | 1.56 | 7 |
|  | protein binding transcription factor activity | 0 | 0 | 0.78 | 8 |
| **Cellular components** | cell | 70.46 | 1 | 71.34 | 1 |
|  | organelle | 46.35 | 2 | 48.89 | 2 |
|  | macromolecular complex | 41.39 | 3 | 38.06 | 3 |
|  | membrane-enclosed lumen | 5.37 | 4 | 7.55 | 4 |
|  | extracellular region | 4.67 | 5 | 6.94 | 5 |
|  | membrane | 3.44 | 6 | 5.28 | 6 |
|  | extracellular matrix | 0.64 | 7 | 0.97 | 7 |
