# Supplementary material for: Genome sequence of Phormia regina Meigen (Diptera: Calliphoridae): implications for medical, veterinary and forensic research
Source: BMC Genomics. 2016 Oct 28;17:842. doi: 10.1186/s12864-016-3187-z (PMC5084420; doi:10.1186/s12864-016-3187-z)
Supplement: Additional file 10: Table S8. — A comparative gene ontology showing the ranking of different functional categories in each species among P. regina (both sexes), M. domestica and D. melanogaster with the rank of 1 indicating the most abundant GO term. (DOC 62 kb) [file 12864_2016_3187_MOESM10_ESM.doc]

Table S8: A comparative gene ontology showing the ranking of different functional categories in each species among *P. regina* (both sexes), *M. domestica* and *D. melanogaster*  with the rank of 1 indicating the most abundant GO term.

|  | | *P. regina* Male | *P. regina* Female | *D. melanogaster* | *M. domestica* |
| --- | --- | --- | --- | --- | --- |
| Biological Processes | cellular process | 1 | 1 | 1 | 2 |
|  | metabolic process | 2 | 2 | 3 | 3 |
|  | single-organism process | 3 | 3 | 2 | 1 |
|  | response to stimulus | 4 | 4 | 8 | 7 |
|  | localization | 5 | 7 | 10 | 10 |
|  | cellular component organization or biogenesis | 6 | 5 | 7 | 8 |
|  | biological regulation | 7 | 8 | 6 | 4 |
|  | signaling | 8 | 6 | 9 | 9 |
|  | biological adhesion | 9 | 13 | - | - |
|  | developmental process | 10 | 9 | 4 | 5 |
|  | growth | 11 | 14 | 12 | 12 |
|  | locomotion | 12 | 12 | - | - |
|  | multicellular organismal process | 13 | 10 | 5 | 6 |
|  | immune system process | 14 | 15 | - | - |
|  | reproduction | 15 | 11 | 11 | - |
| Molecular Function | binding | 1 | 1 | 1 | 1 |
|  | catalytic activity | 2 | 2 | 2 | 2 |
|  | structural molecule activity | 3 | 4 | 4 | 5 |
|  | transporter activity | 4 | 3 | 3 | 3 |
|  | nucleic acid binding transcription factor activity | 5 | 5 | 7 | 7 |
|  | molecular transducer activity | 6 | 6 | 9 | 8 |
|  | molecular function regulator | 7 | 7 | - | - |
|  | transcription factor activity, protein binding | 8 | 8 | - | - |
| Cellular Component | cell | 1 | 1 | 1 | 1 |
|  | organelle | 2 | 2 | 2 | 2 |
|  | macromolecular complex | 3 | 3 | 3 | 3 |
|  | membrane-enclosed lumen | 4 | 6 | 6 | 5 |
|  | extracellular region | 5 | 5 | 4 | 6 |
|  | membrane | 6 | 4 | 5 | 4 |
|  | extracellular matrix | 7 | 7 | 7 | 7 |
