# Supplementary material for: Genome sequence of Phormia regina Meigen (Diptera: Calliphoridae): implications for medical, veterinary and forensic research
Source: BMC Genomics. 2016 Oct 28;17:842. doi: 10.1186/s12864-016-3187-z (PMC5084420; doi:10.1186/s12864-016-3187-z)
Supplement: Additional file 14: Table S11. — Top hits of sex determining genes homologous to D. melanogaster in the male and female P. regina assembled genomes. (DOC 34 kb) [file 12864_2016_3187_MOESM14_ESM.doc]

Table S11. Top hits of sex determining genes homologous to *D. melanogaster* in the male and female *P. regina* assembled genomes.

| ***D. melanogaster* gene** | ***P. regina* contig** | **E-value** | **% identity** | **Hit length (bp)** |
| --- | --- | --- | --- | --- |
| *Transformer** | Female 172,113  Male 124 | 9.30*e*-141  6.46e-145 | 90.74  89.13 | 995  972 |
| *Transformer 2** | Female 174,563  Male 6,339 | 1.60e-19  6.61e-18 | 73.26  72.67 | 172  172 |
| *Sex lethal* | Female 175,544  Male 183,552 | 1.88e-12  1.82e-12 | 82.46  72.36 | 123  123 |
| *Double sex* | Female 187,844  Male 19,892 | 1.18e-42  1.15e-42 | 88.46  71.12 | 329  329 |
| *Fruitless* | Female 16,439  Male 170,850 | 1.04e-68  1.87e-65 | 91.49  75.29 | 412  348 |
| *Daughterless* | Female 172,119  Male 183,755 | 2.17e-73  2.10e-73 | 79.07  79.07 | 371  371 |
| *Maleless* | Female 8,960  Male 167,315 | 9.39e-180  9.10e-180 | 69.54  69.54 | 1211  1211 |

*tBLASTx using other calliphorid genes as queries.
