# Supplementary material for: Genome sequence of Phormia regina Meigen (Diptera: Calliphoridae): implications for medical, veterinary and forensic research
Source: BMC Genomics. 2016 Oct 28;17:842. doi: 10.1186/s12864-016-3187-z (PMC5084420; doi:10.1186/s12864-016-3187-z)
Supplement: Additional file 21: Table S12. — A summary of the top BLASTn results of antimicrobial related genes homologous to Drosophila. The four antimicrobial families are represented by cecropin A1 (cecropin), iconoclast (defensin), hephaestus (diptericin) and relish (attacin). (DOC 37 kb) [file 12864_2016_3187_MOESM21_ESM.doc]

Table S12. A summary of the top BLASTn results of antimicrobial related genes homologous to *Drosophila*. The four antimicrobial families are represented by *cecropin* A1 (cecropin), *iconoclast* (defensin), *hephaestus* (diptericin) and *relish* (attacin).

| ***D. melanogaster* gene** |  | ***P. regina* contig (female)** | **Lowest E-value** | **% identity** | **Hit length (bp)** |
| --- | --- | --- | --- | --- | --- |
| **CG8492 (lysozyme)** |  | 174404 | 5e-80 | 72.13 | 826 |
| ***par-1*** |  | 172054 | 6.14e-58 | 89.33 | 224 |
| ***tlk*** |  | 190533 | 2.8e-96 | 75.6 | 455 |
| ***Gprk2*** |  | 17329 | 1.39e-49 | 75.69 | 255 |
| ***p38a*** |  | 174447 | 0 | 72.65 | 1064 |
| ***CecA 1* (cecropin)** |  | 17009 | 8.87e-10 | 82.35 | 80 |
| ***Ico* (defensin)** |  | 8179 | 0 | 73.91 | 1656 |
| ***Heph (*diptericin*)*** |  | 188192 | 4.86e-161 | 68.36 | 1285 |
| ***Rel (*attacin*)*** |  | 188106 | 6.19e-46 | 69.86 | 482 |
