# Supplementary material for: Genome sequence of Phormia regina Meigen (Diptera: Calliphoridae): implications for medical, veterinary and forensic research
Source: BMC Genomics. 2016 Oct 28;17:842. doi: 10.1186/s12864-016-3187-z (PMC5084420; doi:10.1186/s12864-016-3187-z)
Supplement: Additional file 22: Table S13. — A summary of copy number, number of bases, and percent of the genome derived from repetitive sequences in the female and male Phormia regina genome. Repeats were identified based on homology to known dipteran repetitive sequences in RepBase. (DOC 72 kb) [file 12864_2016_3187_MOESM22_ESM.doc]

Table S13: A summary of copy number, number of bases, and percent of the genome derived from repetitive sequences in the female and male *Phormia regina* genome. Repeats were identified based on homology to known dipteran repetitive sequences in RepBase.

|  | | **Female** | | | **Male** | | |
| --- | --- | --- | --- | --- | --- | --- | --- |
|  |  | **Copy number** | **Bases (bp)** | **% Genome** | **Copy number** | **Bases (bp)** | **% Genome** |
| **Class I Retrotransposons** | | | | | | | |
| LTRs | Copia | 777 | 347,473 | 0.06% | 747 | 333,729 | 0.06% |
|  | Gypsy | 7,252 | 1,583,172 | 0.29% | 7,280 | 1,562,009 | 0.30% |
|  | LTR | 2,894 | 511,383 | 0.09% | 2,510 | 433,920 | 0.08% |
|  | Pao | 2,488 | 592,935 | 0.11% | 2,490 | 591,084 | 0.11% |
| LINEs | CR1 | 1,938 | 380,743 | 0.07% | 1,671 | 317,196 | 0.06% |
|  | Dong-R4 | 13 | 754 | 0.00% | 13 | 751 | 0.00% |
|  | I | 1,344 | 213,070 | 0.04% | 1,200 | 194,310 | 0.04% |
|  | Jockey | 9,612 | 1,894,422 | 0.35% | 8,953 | 1,630,388 | 0.31% |
|  | L1 | 119 | 6,650 | 0.00% | 128 | 6,782 | 0.00% |
|  | L2 | 1,687 | 228,533 | 0.04% | 1,518 | 195,801 | 0.04% |
|  | LOA | 7,920 | 1,229,783 | 0.23% | 7,021 | 1,071,531 | 0.20% |
|  | Penelope | 109 | 30,630 | 0.01% | 123 | 34,306 | 0.01% |
|  | R1 | 3,597 | 700,920 | 0.13% | 3,200 | 609,661 | 0.12% |
|  | R2 | 19 | 5,549 | 0.00% | 23 | 4,954 | 0.00% |
|  | RTE | 275 | 37,722 | 0.01% | 283 | 39,782 | 0.01% |
| SINEs | tRNA | 39 | 2,610 | 0.00% | 39 | 2,665 | 0.00% |
| **Class II DNA Transposons** | | | | | | | |
| Cut and Paste Transposons | CMC | 1,267 | 131,966 | 0.02% | 1,220 | 122,700 | 0.02% |
|  | Crypton | 3 | 213 | 0.00% | 1 | 64 | 0.00% |
|  | hAT | 3,631 | 410,600 | 0.07% | 3,036 | 342,331 | 0.07% |
|  | Kolobok | 27 | 1,611 | 0.00% | 27 | 1,486 | 0.00% |
|  | Merlin | 11 | 1,638 | 0.00% | 11 | 1,804 | 0.00% |
|  | MULE | 1,194 | 72,092 | 0.01% | 49 | 3,074 | 0.00% |
|  | P | 1,084 | 58,452 | 0.01% | 1,061 | 57,686 | 0.01% |
|  | PIF | 996 | 90,459 | 0.02% | 104 | 11,414 | 0.00% |
|  | PiggyBac | 480 | 58,179 | 0.01% | 419 | 51,338 | 0.01% |
|  | Sola | 516 | 32,280 | 0.01% | 457 | 28,756 | 0.01% |
|  | TcMariner | 15,572 | 3,245,817 | 0.60% | 12,404 | 2,502,545 | 0.48% |
|  | Zator | 6 | 331 | 0.00% | 7 | 435 | 0.00% |
|  | Unknown | 2,018 | 320,054 | 0.06% | 1,944 | 309,165 | 0.06% |
| Rolling Circle | Helitron | 22,005 | 2,742,903 | 0.51% | 18,101 | 2,130,585 | 0.41% |
| Unknown | Maverick | 391 | 105,904 | 0.02% | 361 | 96,840 | 0.02% |
| **Other** | | | | | | | |
| *Unknown* | | 1,791 | 122,300 | 0.02% | 1,547 | 104,631 | 0.02% |
| *Low complexity* | | 578,657 | 28,840,267 | 5.32% | 540,356 | 27,411,821 | 5.23% |
| **Total Repeat Content** | | **669,732** | **44,001,415** | **8.11%** | **618,304** | **40,205,544** | **7.68%** |
